# Mortality from drug-resistant tuberculosis in high-burden countries comparing routine drug susceptibility testing with whole-genome sequencing: a multicentre cohort study

**DOI:** 10.1016/S2666-5247(21)00044-6

**Published:** 2021-04-29

**Authors:** Kathrin Zürcher, Martina L Reichmuth, Marie Ballif, Chloé Loiseau, Sonia Borrell, Miriam Reinhard, Veronika Skrivankova, Rico Hömke, Peter Sander, Anchalee Avihingsanon, Alash’le G Abimiku, Olivier Marcy, Jimena Collantes, E Jane Carter, Robert J Wilkinson, Helen Cox, Marcel Yotebieng, Robin Huebner, Lukas Fenner, Erik C Böttger, Sebastien Gagneux, Matthias Egger

**Affiliations:** Institute of Social and Preventive Medicine, University of Bern, Bern, Switzerland (K Zürcher MSc, M L Reichmuth MSc, M Ballif PhD, V Skrivankova PhD, Prof L Fenner MD, Prof M Egger MD); Swiss Tropical and Public Health Institute, Basel, Switzerland (C Loiseau PhD, S Borrell PhD, M Reinhard, Prof S Gagneux PhD); University of Basel, Basel, Switzerland (C Loiseau, S Borrell, M Reinhard, Prof S Gagneux); Institute of Medical Microbiology, University of Zurich, Zurich, Switzerland (R Hömke, P Sander MD, Prof E C Böttger MD); Swiss National Center for Mycobacteria, Zurich, Switzerland (R Hömke, P Sander, Prof E C Böttger); The HIV Netherlands Australia Thailand Research Collaboration, Thai Red Cross AIDS Research Centre and Tuberculosis Research Unit, Faculty of Medicine, Chulalongkorn University, Bangkok, Thailand (A Avihingsanon MD); Institute of Human Virology Nigeria, Abuja, Nigeria (Prof A G Abimiku PhD); Centre de Prise en Charge de Recherche et de Formation, Yopougon, Abidjan, Côte d’Ivoire (O Marcy MD); Instituto de Medicina Tropical Alexander von Humboldt, Universidad Peruana Cayetano Heredia, Lima, Peru (J Collantes MSc); Department of Medicine, Moi University School of Medicine, and Moi Teaching and Referral Hospital, Eldoret, Kenya (Prof E J Carter MD); Brown University School of Medicine, Providence, RI, USA (Prof E J Carter); Wellcome Centre for Infectious Diseases Research in Africa (Prof R J Wilkinson PhD, Prof H Cox PhD), Institute for Infectious Disease and Molecular Medicine (Prof H Cox), and Centre for Infectious Disease Epidemiology and Research, Faculty of Health Sciences (Prof M Egger), University of Cape Town, Cape Town, South Africa; Department of Infectious Diseases, Imperial College London, London, UK (Prof R J Wilkinson); The Francis Crick Institute, London, UK (Prof R J Wilkinson); National TB Lab, Kinshasa, Democratic Republic of the Congo (Prof M Yotebieng MD); Albert Einstein College of Medicine, New York, NY, USA (Prof M Yotebieng); National Institutes of Allergy and Infectious Diseases, National Institutes of Health, Bethesda, MD, USA (R Huebner PhD)

## Abstract

**Background:**

Drug resistance threatens global tuberculosis control. We aimed to examine mortality in patients with tuberculosis from high-burden countries, according to concordance or discordance of results from drug susceptibility testing done locally and whole-genome sequencing (WGS).

**Methods:**

In this multicentre cohort study, we collected pulmonary *Mycobacterium tuberculosis* isolates and clinical data from individuals with tuberculosis from antiretroviral therapy programmes and tuberculosis clinics in Côte d’Ivoire, Democratic Republic of the Congo, Kenya, Nigeria, Peru, South Africa, and Thailand, stratified by HIV status and drug resistance. Sites tested drug susceptibility using routinely available methods. WGS was done on Illumina HiSeq 2500 in the USA and Switzerland, and TBprofiler was used to analyse the genomes. We included individuals aged 16 years or older with pulmonary tuberculosis (bacteriologically confirmed or clinically diagnosed). We analysed mortality in multivariable logistic regression models adjusted for sex, age, HIV status, history of tuberculosis, and sputum positivity.

**Findings:**

Between Sept 1, 2014, and July 4, 2016, of 634 patients included in our previous analysis, we included 582 patients with tuberculosis (median age 33 years [IQR 27–43], 225 [39%] women, and 247 [42%] HIV-positive). Based on WGS, 339 (58%) isolates were pan-susceptible, 35 (6%) monoresistant, 146 (25%) multidrug-resistant, and 24 (4%) pre-extensively drug-resistant (pre-XDR) or XDR. The analysis of mortality was based on 530 patients; 63 (12%) died and 77 (15%) patients received inappropriate treatment. Mortality ranged from 6% (18 of 310) in patients with pan-susceptible tuberculosis to 39% (nine of 23) in patients with pre-XDR or XDR tuberculosis. The adjusted odds ratio for mortality was 4·92 (95% CI 2·47–9·78) among undertreated patients, compared with appropriately treated patients.

**Interpretation:**

In seven countries with a high burden of tuberculosis, we observed discrepancies between drug resistance patterns obtained locally and WGS. The underdiagnosis of drug resistance resulted in inappropriate treatment and higher mortality. WGS can provide accurate and detailed drug resistance information required to improve the outcomes of drug-resistant tuberculosis in high-burden settings. Our results support WHO’s call for point-of-care tests based on WGS.

**Funding:**

National Institutes of Allergy and Infectious Diseases, Swiss National Science Foundation, and Swiss National Center for Mycobacteria.

## Introduction

Tuberculosis is caused by bacteria of the *Mycobacterium tuberculosis* complex and is the leading cause of death by a single infectious agent worldwide.1 In 2019, ten million people were estimated to have developed active tuberculosis, of whom 8% also had HIV. In the same year, around 1·2 million people died from tuberculosis, including 208 000 people with HIV.^1^ Tuberculosis accounts for approximately 40% of HIV and AIDS-related adult deaths, and half of these remain undiagnosed.^2^

The emergence of drug-resistant *M tuberculosis* strains threatens tuberculosis control. In 2019, 3% of new tuberculosis cases worldwide were estimated to be multidrug-resistant (MDR) tuberculosis, and 18% of individuals who had been previously treated had MDR tuberculosis.1 People with HIV are at greater risk of acquiring MDR tuberculosis than people who are HIV-negative.^3^ Also, treatment outcomes in people with HIV and MDR tuberculosis are worse than among HIV-negative patients with MDR tuberculosis.3 Pre-extensively drug-resistant (pre-XDR) or XDR tuberculosis poses additional challenges for treatment and control of the disease.^4^ Strategies to control and prevent drug-resistant tuberculosis include surveillance, rapid drug susceptibility testing, and ensuring the completion of an appropriate treatment regimen. The limited access to detailed drug susceptibility testing and effective second-line anti-tuberculosis drugs, insufficient adherence and drug dosages, and comorbidities challenge the management of drug-resistant tuberculosis in low-income and middle- income countries.^2,5–7^

The present study is part of a research programme investigating drug-resistant tuberculosis of the International epidemiology Databases to Evaluate AIDS.^8^ In a previous analysis, we compared the results of drug susceptibility testing from high-burden countries in Africa, Asia, and Latin America with phenotypic drug susceptibility testing results from the Swiss National Center for Mycobacteria.^9^ We found that the accuracy of testing done at participating sites was moderate, and that discordant results and inappropriate treatment were associated with increased mortality. The Swiss reference laboratory tested drug resistance to six drugs only: isoniazid, rifampicin, pyrazinamide, ethambutol, amikacin, and moxifloxacin. Therefore, other resistances could have been missed, including resistance to streptomycin, kanamycin, ethionamide, levofloxacin, or newer drugs.

Whole-genome sequencing (WGS) can simultaneously provide information on resistance to first-line and second-line drugs, for which drug-resistance-conferring mutations are known. WGS has the potential to overcome many of the limitations of conventional drug susceptibility testing with higher throughput.10 We and others showed that drug susceptibility predicted from *M tuberculosis* genomes correlates with phenotypic drug susceptibility testing.^11,12^ WHO recommends WGS for drug resistance surveillance and is evaluating sequencing technologies for routine drug susceptibility testing. ^1,13^ Here, we aimed to compare the drug resistance patterns routinely obtained in seven countries with a high tuberculosis burden with the results from WGS, and examined the mortality associated with discordant resistance profiles using WGS as the reference.

## Methods

### Study design and participants

We did a multicentre cohort study. As described in detail elsewhere, ^9^ we recruited patients from antiretroviral therapy programmes and tuberculosis clinics in their corresponding catchment areas in Côte d’Ivoire, Democratic Republic of the Congo, Kenya, Nigeria, Peru, South Africa, and Thailand. In South Africa, we used strain collections held at the University of Cape Town (Cape Town, South Africa). All patients had bacteriologically confirmed, or clinically diagnosed tuberculosis. We included individuals aged 16 years or older with pulmonary tuberculosis. We excluded patients for whom no viable isolate was available, patients with extrapulmonary tuberculosis only, patients with missing data that were necessary for the analyses, and patients for whom the *M tuberculosis* genome could not be sequenced (appendix p 2). Recruitment was stratified by HIV status and drug resistance as defined at local clinics. We collected demographic and clinical characteristics of participants using a standardised questionnaire. *M tuberculosis* isolates were subcultured at the recruitment sites.

The Cantonal Ethics Committee in Bern, Switzerland, and local institutional review boards approved the study. Written informed consent was obtained at all sites, except in South Africa, where consent was not required for the use of archived samples.

### Procedures

The local laboratories tested molecular or phenotypic drug susceptibility according to routine procedures. DNA was extracted from isolates using standard protocols.^14^ Libraries were prepared using the Illumina Nextera XT kit (Illumina, San Diego, CA, USA) and sequenced on Illumina HiSeq 2500 at the Department of Biosystems Science and Engineering of the Swiss Federal Institute of Technology in Basel, Switzerland and the Broad Institute in Cambridge, MA, USA. Sequences had 101, 138, or 151 bp paired-end reads. After Illumina adaptors were clipped and low-quality reads trimmed with Trimmomatic, version 0.38, reads shorter than 36 bp were excluded. The minimum read depth at each position was 10 × in 99% of the genome (IQR 99–99, range 77–100; seven genomes were less than 90%). BCFtools, version 1.11 mpileup was used to map the reads to the H37Rv reference genome. We included reads with a minimum mapping quality of eight. We screened one isolate per patient for anti-tuberculosis drug resistance mutations using the TBprofiler, version 2.8.2 pipeline.^10,15^ The pipeline aligns reads to the reference genome using BWA, version 0.7.17 and calls variants with SAMtools, version 1.9.^10,16–18^ The variants were then compared to a drug resistance database. Single-nucleotide polymorphisms, insertions, and deletions responsible for resistance to 19 anti-tuberculosis drugs were identified:^10,15,19^ streptomycin, para-aminosalicylic acid, isoniazid, pyrazinamide, cycloserine, kanamycin, ethionamide, ethambutol, amikacin, rifampicin, capreomycin, ofloxacin, ciprofloxacin, moxafloxacin, levofloxacin, linezolid, bedaquiline, clofazimine, and delamanid. A coverage of ten reads was needed to call a polymorphism. We considered all drug resistance alleles with a variant frequency equal to or higher than 90%.

WHO defines monoresistance as resistance to one of the first-line drugs (ie, isoniazid, pyrazinamide, ethambutol, and rifampicin).^1,13^ MDR tuberculosis is defined as resistance to both isoniazid and rifampicin. Pre-XDR tuberculosis is defined as resistance to isoniazid and rifampicin plus fluoroquinolones or one of the three second-line injectable drugs (ie, amikacin, ciprofloxacin, or kanamycin). XDR tuberculosis is defined as drug resistance against isoniazid, rifampicin, fluoroquinolones, and at least one of the three second-line injectable drugs.

We compared the drug resistance profiles obtained at sites using routine drug susceptibility testing to drug resistance patterns obtained from whole-genome sequences. We considered any drug resistance obtained from the tests that a patient underwent locally. Drug resistance profiles were defined as concordant or discordant according to the resistance categories defined by WHO.^1^ Discordant results were further categorised into discordant results potentially leading to undertreatment, or potentially leading to overtreatment (appendix p 6).^1,13^ Discordances with no clear implications for treatment were defined as other discordances. We assessed the appropriateness of prescribed anti- tuberculosis treatment according to WHO guidelines (appendix p 7).^1,13^ Effective drugs were defined as drugs to which no drug-resistance-conferring mutations were observed in WGS (appendix p 8). The prescription of less than three effective drugs was defined as undertreatment, except for patients with isoniazid-resistant or rifampicin- resistant isolates. In these patients, a regimen comprising fewer than four effective drugs was considered as undertreatment, according to WHO guidelines. Overtreatment included second-line drugs given to patients for whom first-line regimens would have been appropriate. The classification of regimens is shown in the appendix (p 11).

### Statistical analysis

We used descriptive statistics for patient characteristics by levels of drug resistance based on WGS. We compared the following drug resistance categories: pan-susceptible tuberculosis, monoresistant tuberculosis (any monoresistance), MDR tuberculosis, pre-XDR or XDR tuberculosis, any isoniazid-resistant tuberculosis (including isoniazid-monoresistant, MDR, and pre-XDR or XDR tuberculosis), any rifampicin-resistant tuberculosis (including rifampicin-monoresistant, MDR, and pre-XDR or XDR tuberculosis). Patients with missing data for treatment regimen, treatment outcome, ongoing treatment, or sputum microscopy were excluded from the analysis of mortality.

Four logistic regression models were calculated to assess the effects of: any drug resistance; drug resistance categories; discordant diagnoses; and treatment appropriateness on mortality. Logistic regression models were adjusted for sex, age, HIV status, history of tuberculosis, and sputum positivity. The country of origin was included as a random effect on the intercept.^20^ We did three sensitivity analyses. First, we repeated all logistic regression analyses after restricting the data to drug resistances that could be diagnosed with the locally available tests. We thus excluded drug resistances that were missed due to unavailable testing methods. Second, we repeated the logistic regression for mortality by treatment appro priateness, excluding patients with pre-XDR or XDR tuberculosis. Third, we examined the effect of different variant frequency cutoffs on each logistic regression (≥0% and 100%). All analyses were done in R, version 3.6.1, or Python, version 3.7.6.^21,22^

### Role of the funding source

The funders of the study had no role in study design, data collection, data analysis, data interpretation, or writing of the report.

## Results

Between Sept 1, 2014, and July 4, 2016, of the 634 patients included in our previous analysis,^9^ we were unable to sequence 52 (8%) isolates due to poor bacterial growth, DNA quality, or failures in the library preparation (appendix p 2). We therefore included 582 patients with tuberculosis, 406 (70%) from Africa, 93 (16%) from Latin America, and 83 (14%) from Asia. 172 (30%) patients came from South Africa, 94 (16%) from Côte d’Ivoire, 93 (16%) from Peru, 83 (14%) from Thailand, 59 (10%) from Democratic Republic of the Congo, 53 (9%) from Nigeria, and 28 (5%) from Kenya (table 1). The median age was 33 years (IQR 27–43), 225 (39%) were women, and 247 (42%) were HIV-positive. Six *M tuberculosis* lineages were represented: 24 (4%) cases of L1, 135 (23%) L2, 18 (3%) L3, 403 (69%) L4, one (<1%) L5, and one (<1%) L6.

**Figure 1:**
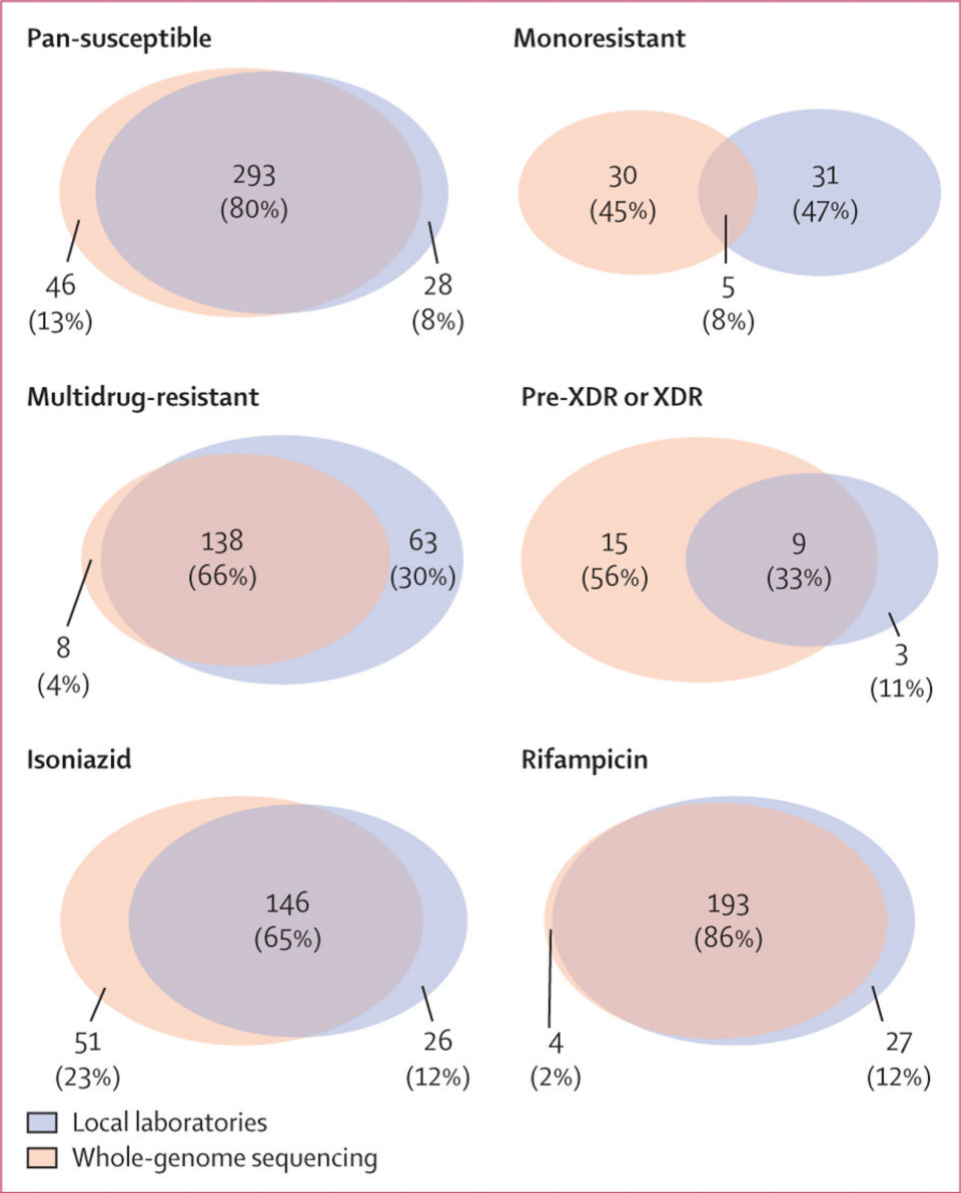
Distribution of diagnosed drug resistance between whole-genome sequencing and local drug susceptibility testing The categories include pan-susceptible, monoresistant (any monoresistance), multidrug-resistant, pre-XDR or XDR, any isoniazid-resistant, or any rifampicin-resistant tuberculosis. Due to rounding, some group percentage totals are more than 100%. XDR=extensively drug-resistant.

Based on WGS, 339 (58%) isolates were pan-susceptible and 35 (6%) were monoresistant: 24 rifampicin, eight isoniazid, two pyrazinamide, and one ethambutol mono- resistant isolates. There were 208 (36%) polyresistant isolates, including 146 (25%) MDR, 24 (4%) pre-XDR or XDR isolates, and 38 (7%) other types of polyresistances (table 1; figure 1). Among the 24 patients with pre-XDR or XDR, nine had resistance to fluoroquinolones, six to injectable drugs, and nine to both.

Local drug susceptibility testing results were based on the molecular Xpert MTB/RIF test system, line probe assays, and culture-based phenotypic tests, or a combination of these methods (table 2). Among the 582 isolates, 130 (22%) of 582 had discordant drug resistance results when comparing local drug susceptibility testing with WGS. 65 (11%) discordant drug resistance results potentially led to inappropriate treatment of patients with tuberculosis (table 2). We then looked at the regimens prescribed to patients. For six patients, we had no treatment information. Of 576 patients with known treatment, we observed that overall 86 (15%) of 576 patients received inappropriate treatment according to WGS results and WHO treatment guidelines: 67 (12%) of 576 patients were undertreated, and 19 (3%) were overtreated. Consequently, 490 (85%) patients were appropriately treated.

The agreement between local drug susceptibility testing and WGS was 80% for pan-susceptible, 8% for monoresistant, 66% for MDR, and 33% for pre-XDR or XDR tuberculosis (figure 1). Agreement of local drug susceptibility testing and WGS for rifampicin resistance was 86% and it was 65% for isoniazid resistance. Rifampicin resistance was, in contrast to other drug resistance, more frequently diagnosed with local drug susceptibility testing than with WGS (figure 1). Only three sites tested for drugs other than rifampicin and isoniazid. Two sites tested for streptomycin, two for fluoroquinolones, and two for injectable drugs. One site tested for pyrazinamide and one site for ethambutol. Resistance to pyrazinamide, cycloserine, ethambutol, linezolid, bedaquiline, clofazimine, and delamanid was not tested at any site. WGS did not identify any resistance to bedaquiline, clofazimine, or delamanid (appendix p 8).

**Figure 2:**
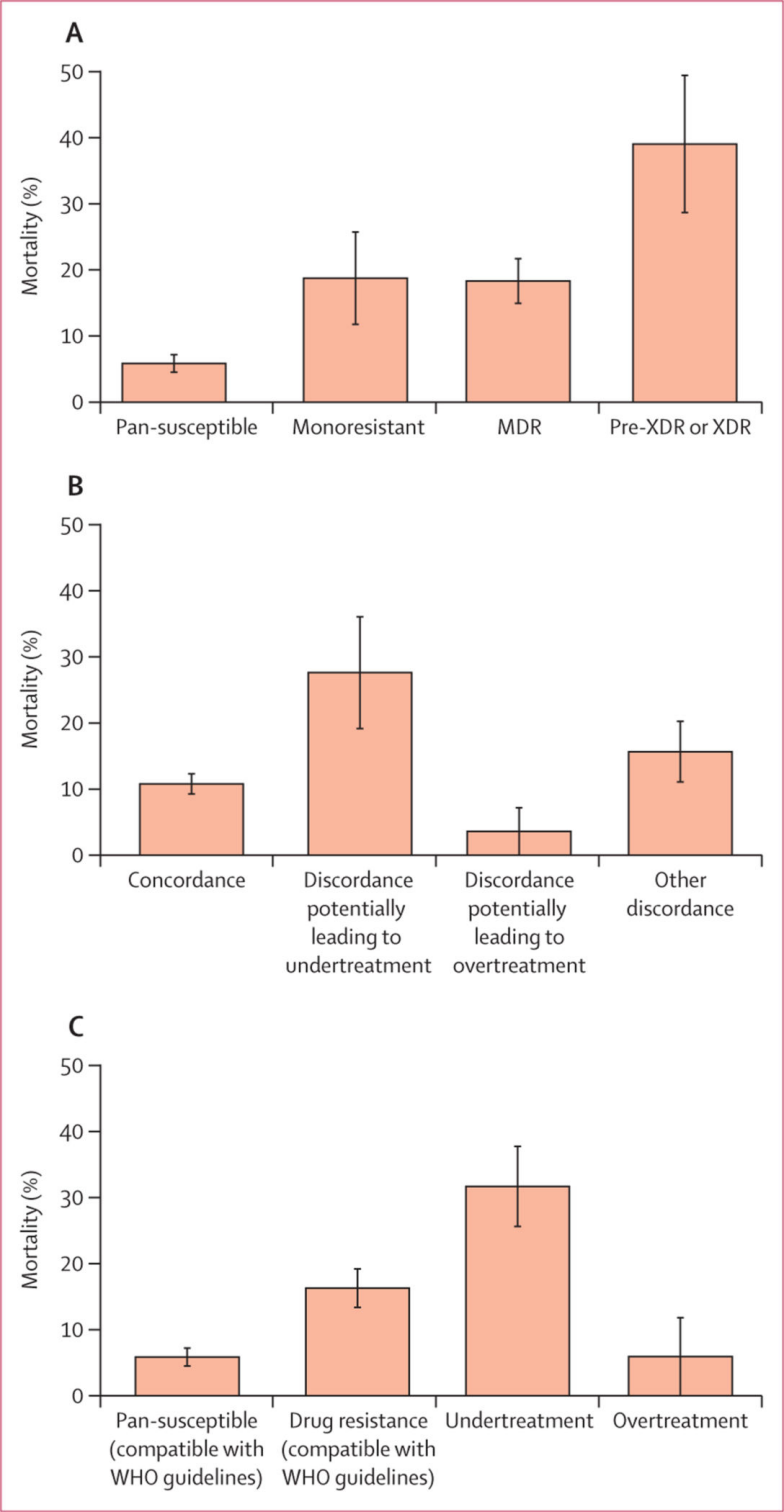
Mortality according to drug resistance, concordance of diagnosis, and treatment appropriateness Mortality data are shown based on drug resistance (A), concordance of diagnosis (B), and treatment appropriateness (C). Appropriateness was considered according to WHO guidelines (appendix pp 6–7). Error bars are SEs. Analysis based on 530 patients with complete data. Mortality was calculated by dividing deaths by the number of patients in the respective category. MDR=multidrug-resistant. XDR=extensively drug-resistant.

We excluded 52 (9%) of 582 patients from the mortality analyses due to missing data (appendix p 2). Based on WGS, the isolates of 310 (58%) of 530 patients were pan-susceptible, 32 (6%) monoresistant, 131 (25%) MDR, 23 (4%) pre-XDR or XDR, and 34 (6%) other polyresistances. Among the 530 patients, 121 (23%) had discordant drug susceptibility testing results. For 29 (66%) of 44 patients, underdiagnosis of drug resistance potentially led to undertreatment, and for 28 (36%) of 77, overdiagnosis potentially led to overtreatment. During treatment, 63 (12%) of 530 patients died (table 3). Mortality was 6% (18 of 310) in patients with pan-susceptible tuberculosis, 19% (six of 32) in patients with monoresistant tuberculosis, and 18% (24 of 131) in patients with MDR tuberculosis. Patients with pre-XDR or XDR tuberculosis had a mortality of 39% (nine of 23; figure 2A). Overall, mortality ranged from 6% (16 of 267) among patients with pan-susceptible strains and concordant diagnosis to 47% (seven of 15) among patients with pre-XDR or XDR tuberculosis and a discordant diagnosis potentially leading to undertreatment (table 3). In patients with a discordant diagnosis potentially leading to undert- reatment, mortality was 28% (eight of 29), and in patients with a discordant diagnosis potentially leading to overtreatment, it was 4% (one of 28; figure 2B). Mortality ranged from 6% (17 of 293) in patients with pan-susceptible tuberculosis treated according to WHO guidelines to 32% (19 of 60) in undertreated patients and 6% (one of 17) in patients who were overtreated (figure 2C).

**Figure 3:**
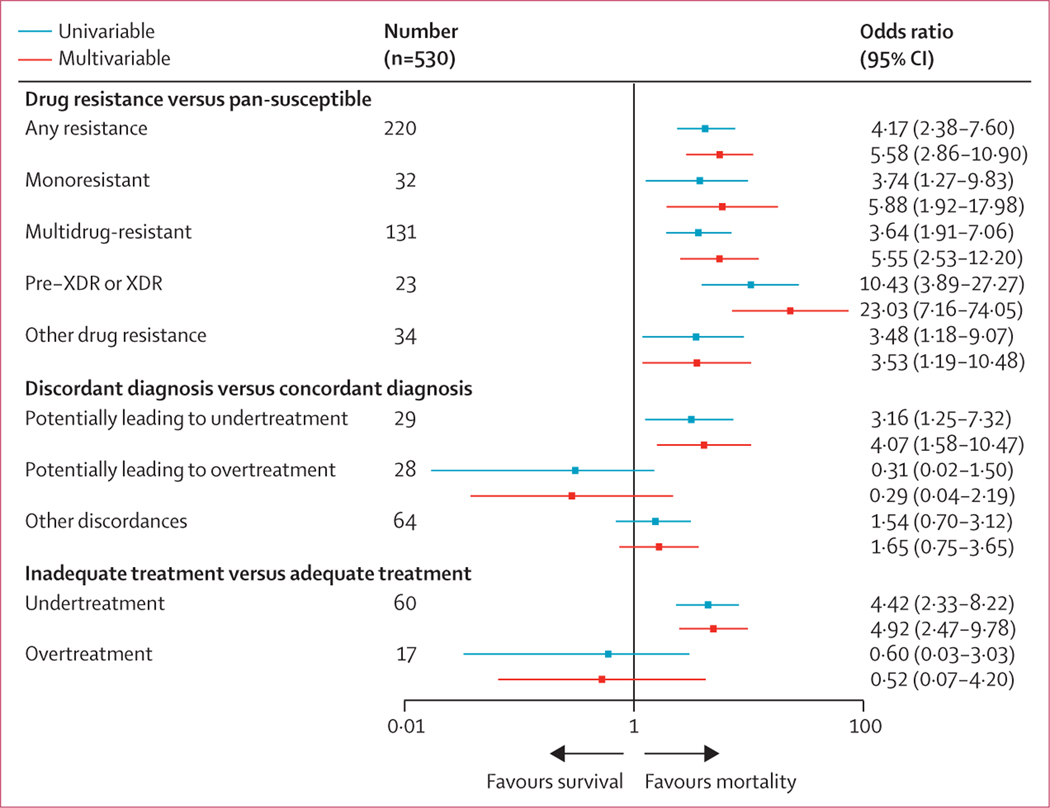
Logistic regression models to assess the effect of any drug resistance, drug resistance categories, diagnosis discordance, and treatment appropriateness on mortality The models were adjusted for sex, age, HIV status, history of tuberculosis, and sputum microscopy, and country of participating site was included as random effect on the intercept. Appropriateness was considered according to WHO guidelines (appendix pp 6–7). XDR=extensively drug-resistant.

In the multivariable logistic regression, resistance to any of the anti-tuberculosis drugs was associated with higher mortality (figure 3). The adjusted odds ratio (OR) was 5·58 (95% CI 2·86–10·90). The association with mortality became stronger with a higher degree of drug resistance. Compared with pan-susceptible tuberculosis, the adjusted OR for monoresistant was 5·88 (95% CI 1·92–17·98), for MDR was 5·55 (2·53–12·20), and for pre-XDR or XDR tuberculosis was 23·03 (7·16–74·05; figure 3). The adjusted OR for mortality during tuberculosis treatment was 4·07 (95% CI 1·58–10·47) in patients with a diagnosis potentially leading to undertreatment, and 0·29 (0·04–2·19) in the case of a diagnosis potentially leading to overtreatment, compared with patients with appropriate treatment (figure 3). Overall, 77 (15%) of 530 patients received inappropriate treatment based on WGS drug resistance results and WHO guidelines (appendix p 7). 60 (11%) of 530 patients were undertreated, and 17 (3%) of 530 were overtreated. The OR for mortality for undertreatment was 4·92 (95% CI 2·47–9·78), and for overtreatment was 0·52 (0·07–4·20), compared with patients receiving appropriate treatment (figure 3). In a sensitivity analysis, we showed that mortality among undertreated patients remained higher than among appropriately treated patients after excluding patients with pre-XDR or XDR tuberculosis (adjusted OR 5·97 [95% CI 2·58–13·80]). The unadjusted covariate ORs for mortality during tuberculosis treatment are shown in the appendix (p 13). The sensitivity analysis of the logistic regression models using different variant frequency cutoffs (≥0% and 100%) produced similar results (appendix pp 3–4). When restricting the analysis to drug resistances that could be diagnosed at sites, again similar results were obtained (appendix p 5).

## Discussion

In this multicentre cohort study, we compared drug resistance predicted by WGS with the results from local drug susceptibility testing in seven countries with a high burden of tuberculosis. We examined mortality by drug resistance predicted by WGS, and by concordance or discordance with local diagnosis and the appropriateness of treatment. We found that the diagnosis was discordant between local drug resistance results and WGS in about one in five patients. The agreement between local and centralised WGS was the highest for rifampicin and isoniazid, but low for other drugs. Of note, resistance to streptomycin, para-aminosalicylic acid, pyrazinamide, cycloserine, ethionamide, ethambutol, fluoroquinolones, and injectable drugs was rarely investigated locally. Mortality during treatment ranged from 6% among patients with pan-susceptible strains and concordant results between WGS and local drug resistance testing to 47% among patients with pre-XDR or XDR tuberculosis and discordant results.

To our knowledge, this is the first study to compare the results from drug susceptibility testing in real-world settings in high-burden countries with WGS and to examine the effect of discordant resistance results on mortality. In a previous analysis of this cohort, we compared the results from local drug susceptibility testing with those obtained at the Swiss National Center for Mycobacteria for six drugs.^9^ In the present study, we used a well established bioinformatics pipeline and its corresponding database to analyse the WGS data.^10^ The analysis covered 19 anti-tuberculosis drugs, including streptomycin, kanamycin, pyrazinamide, ethionamide, ethambutol, and levofloxacin, as well as newer drugs. Specifically, we were able to detect more single-drug resistance with WGS than with phenotypic drug susceptibility testing.

Rapid and accurate diagnosis, prompt and appropriate treatment, and the control of airborne infection are key strategies to prevent drug-resistant tuberculosis ^23^ Routine testing at sites focused mainly on the identification of rifampicin and isoniazid resistance used to diagnose MDR tuberculosis and did not address the efficacy of other drugs. Also, isoniazid monoresistance would typically be missed if drug susceptibility testing relies on the Xpert MTB/RIF system, which could lead to the undertreatment of some patients. Furthermore, culture-based drug susceptibility testing is challenging for several drugs—eg, pyrazinamide, ethionamide, and ethambutol— due to poor drug solubility.^11,24^ Yet, pyrazinamide is essential for shortening tuberculosis therapy, and resistance to pyrazinamide is associated with worse outcomes.^23^ However, pyrazinamide resistance testing is often unavailable. Only one site could test pyrazinamide resistance in our study.

WGS has the potential to predict resistance profiles for most anti-tuberculosis drugs without the need for time- consuming phenotypic drug susceptibility testing.^10,12–19^ WGS provides simultaneous and comprehensive information on relevant mutations conferring resistance to first-line and second-line drugs, anywhere in the genome. By contrast, targeted sequencing only identifies mutations in a priori defined regions covered by the amplifications. WGS allows effective individualised treatment, and thus reduces the risk of propagating drug resistance. Ineffective treatment could lead to the acquisition of additional drug resistance and increases the risk of transmitting drug-resistant strains.23 These considerations support the use of WGS to replace the current drug susceptibility testing methods, which cover only a limited number of drugs.

The broader range of drug resistance captured by WGS explains some of the discordant results found in this study; however, restricting the analysis of discordances between drug resistance diagnosed locally and by WGS to the most clinically relevant WHO categories of drug resistance will have minimised this effect.^13^ Thus, discordant results potentially leading to inappropriate treatment were mainly due to important drug resistance not captured with the available local tests at sites, rather than to a wider range of drug resistances captured by WGS. The detection of drug resistance is also influenced by the type of sample collected, and the methods used for culturing, DNA extraction and sequencing, and the pipeline used to analyse the sequences.^25^ The pipeline used to analyse the sequences was determined by a 90% or greater variant frequency cutoff, the robustness of the TBprofiler pipeline, and its coverage of all relevant resistance-conferring mutations. Our sensitivity analysis showed that the cutoff for variant frequency had little effect on results.

For new drugs, most resistance-conferring mutations are unknown at the time of introduction, and relevant drug resistance mechanisms become apparent only when the mutation becomes established in the population. The TBprofiler database is continuously updated with newly identified resistance-conferring mutations, such as bedaquiline in 2013 and dalamanid in 2014. Yet, the accuracy of the prediction of phenotypic resistance by molecular markers varies by drugs, depending on the molecular mechanisms involved and the evidence generated so far. We showed that the identification of drug-resistance-conferring mutations predicted phenotypic resistance to rifampicin better than to ethambutol.^11^ Discrepancies in results between local drug susceptibility testing and WGS might also be explained by mixed infections, heteroresistance, minority resistant populations, or methodological differences,^25–27^ which can lead to uncertainties in treatment decisions.^28^ Of note, overtreatment did not increase mortality, but the analysis was based on few patients (n=28) and should be interpreted with caution. Anti-tuberculosis drugs, especially second-line drugs, can cause serious side-effects, which can lead to treatment interruption, and failure, or acquired drug resistance, and should therefore only be used when needed.^29^

Our study has several limitations. We sampled eligible patients within strata defined by drug resistance and HIV infection, and therefore, could not estimate the incidence or prevalence of drug-resistant tuberculosis in patients who were HIV-coinfected or HIV-negative. Also, we could not evaluate differences in drug resistance between *M tuberculosis* lineages because the sample size was small for several lineages. Our analysis is mainly based on L2 and L4 strains, as expected from the geographical distribution of these lineages.^30^ Further, we sequenced strains before treatment and thus could not diagnose potentially acquired drug resistance, which might influence treatment outcomes. Finally, this study reflects the years 2013–16. Since then, the availability of drug resistance tests has increased (appendix p 14). For example, the MTBDRsl assay (Hain Lifescience, Nehren, Germany), a line probe assay for the detection of pre-XDR or XDR, is now available at four sites. However, three of the seven sites still have no access to rapid molecular tests to diagnose resistance to second-line drugs. In general, there were only a few changes in the drug resistances that are tested routinely between the study period and 2020 (appendix p 14).

Treatment guidelines also changed over the study period. In 2013, WHO published an interim policy guideline on bedaquiline, and in 2014 on delamanid in the treatment of MDR tuberculosis.^31,32^ In our study, patients were rarely given newer drugs such as bedaquiline or delamanid. In 2020, only South Africa included bedaquiline in their short and long MDR tuberculosis regimens. By contrast, the other sites are still using the so-called Bangladesh regimen (ie, a standardised short course MDR tuberculosis treatment regimen of 9–12 months), although guidelines will probably change in the near future. Identifying the emergence of resistance to recently introduced drugs will be crucial alongside the roll-out of new regimens.^33^

Our study shows that treatment strategies guided by comprehensive drug resistance data are likely to save lives. Our results thus support WHO’s call for an accurate point- of-care test based on WGS that can be done directly from sputum samples.^34^ Such tests would allow rapid diagnosis and efficient, individual-based treatment of drug-resistant tuberculosis.^35^ Test systems performing WGS on sputum samples, using new laboratory and bioinformatics pipelines are in development. High-burden countries should consider building central, high-throughput sequencing capacities.^36^ The establishment of a trustworthy, widely accepted drug resistance database similar to the Stanford HIV drug resistance database will be essential in this context.^37^ Finally, we support the call for clinical trials evaluating the safety, efficacy, and tolerability of new drugs and drug susceptibility testing strategies for drug-resistant tuberculosis.^23,29^ The role of new drugs like bedaquiline, delamanid, and pretomanid in regimens with fewer, more effective, and safer drugs needs to be evaluated.^23^ Future studies should also examine treatment duration and adherence.^23^ The duration of the intensive and continuation phases of tuberculosis treatment and treatment adherence are crucial for efficient therapy.

In conclusion, our study shows that both the accuracy of drug susceptibility testing in routine care, and the access to testing for resistance for several essential drugs is limited in high-burden tuberculosis countries, which leads to inappropriate treatment, and contributes to higher mortality. Our results support the role of WGS to improve the management of drug-resistant tuberculosis in high-burden settings.

## Supplementary Material

1

## Figures and Tables

**Table 1:** Patient characteristics by resistance profiles obtained by whole-genome sequencing

	Pan-susceptible	Any resistance	p value	Monoresistance					Polyresistance			
				All	Isoniazid	Pyrazinamide	Ethambutol	Rifampicin	All	Multidrug-resistant	Pre-XDR or XDR	Other
Total	339	243	··	35	8	2	1	24	208	146	24	38
Sex	··	··	0·99	··	··	··	··	··	··	··	··	··
Women	131 (39%)	94 (39%)	··	10 (29%)	3 (38%)	0 (0%)	1 (100%)	6 (25%)	84 (40%)	56 (38%)	13 (54%)	15 (39%)
Men	208 (61%)	149 (61%)	··	25 (71%)	5 (63%)	2 (100%)	0	18 (75%)	124 (60%)	90 (62%)	11 (46%)	23 (61%)
Age, years	··	··	0·0067	··	··	··			··	··	··	··
At diagnosis	35 (28–45)	32 (25–40)	··	32 (25–40)	40 (31–49)	26 (25–28)	36 (36–36)	29 (25–39)	32 (26–40)	31 (25–39)	30 (25–34)	36 (29–44)
HIV status	··	··	<0·0001	··	··	··	··	··	··	··	··	··
HIV-negative	169 (50%)	166 (68%)	··	23 (66%)	7 (88%)	1 (50%)	0	15 (63%)	143 (69%)	103 (71%)	14 (58%)	26 (68%)
HIV-positive	170 (50%)	77 (32%)	··	12 (34%)	1 (13%)	1 (50%)	1 (100%)	9 (38%)	65 (31%)	43 (29%)	10 (42%)	12 (32%)
*Mycobacterium tuberculosis* lineage	··	··	0·039	··	··	··	··	··	··	··	··	··
L1	18 (5%)	6 (2%)	··	2 (6%)	0	0	0	2 (8%)	4 (2%)	1 (1%)	0	3 (8%)
L2	79 (23%)	56 (23%)	··	7 (20%)	3 (38%)	1 (50%)	0	3 (13%)	49 (24%)	23 (16%)	8 (33%)	18 (47%)
L3	15 (4%)	3 (1%)	··	0	0	0	0	0	3 (1%)	2 (1%)	1 (4%)	0
L4	225 (66%)	178 (73%)	··	26 (74%)	5 (63%)	1 (50%)	1 (100%)	19 (79%)	152 (73%)	120 (82%)	15 (63%)	17 (45%)
L5	1 (<1%)	0	··	0	0	0	0	0	0	0	0	0
L6	1 (<1%)	0	··	0	0	0	0	0	0	0	0	0
Country	··	··	<0·0003		··	··	··	··	··	··	··	··
Côte d’Ivoire	46 (14%)	48 (20%)	··	5 (14%)	2 (25%)	0	1 (100%)	2 (8%)	43 (21%)	39 (27%)	3 (13%)	1 (3%)
Democratic Republic of the Congo	29 (9%)	30 (12%)	··	1 (3%)	0	0	0	1 (4%)	29 (14%)	19 (13%)	8 (33%)	2 (5%)
Kenya	21 (6%)	7 (3%)	··	1 (3%)	1 (13%)	0	0	0	6 (3%)	5 (3%)	0	1 (3%)
Nigeria	19 (6%)	34 (14%)	··	6 (17%)	0	0	0	6 (25%)	28 (13%)	20 (14%)	4 (17%)	4 (11%)
Peru	57 (17%)	36 (15%)	··	2 (6%)	2 (25%)	0	0	0	34 (16%)	28 (19%)	2 (8%)	4 (11%)
South Africa	111 (33%)	61 (25%)	··	15 (43%)	0	1 (50%)	0	14 (58%)	46 (22%)	28 (19%)	7 (29%)	11 (29%)
Thailand	56 (17%)	27 (11%)	··	5 (14%)	3 (38%)	1 (50%)	0	1 (4%)	22 (11%)	7 (5%)	0	15 (39%)
History of tuberculosis	··	··	<0·0001	··	··	··	··	··	··	··	··	··
No	269 (79%)	104 (43%)	··	13 (37%)	7 (88%)	1 (50%)	1 (100%)	4 (17%)	91 (44%)	56 (38%)	5 (21%)	30 (79%)
Yes	70 (21%)	139 (57%)	··	22 (63%)	1 (13%)	1 (50%)	0	20 (83%)	117 (56%)	90 (62%)	19 (79%)	8 (21%)
Treatment outcomes	··	··	<0·0001	··	··	··	··	··	··	··	··	··
Success	248 (73%)	129 (53%)	··	16 (46%)	5 (63%)	0	1 (100%)	10 (42%)	113 (54%)	76 (52%)	11 (46%)	26 (68%)
Mortality	19 (6%)	45 (19%)	··	6 (17%)	1 (13%)	1 (50%)	0	4 (17%)	39 (19%)	24 (16%)	9 (38%)	6 (16%)
Treatment failure	11 (3%)	10 (4%)	··	3 (9%)	0	1 (50%)	0	2 (8%)	7 (3%)	5 (3%)	2 (8%)	0
Lost to follow-up	26 (8%)	29 (12%)	··	5 (14%)	0	0	0	5 (21%)	24 (12%)	22 (15%)	0	2 (5%)
Transfer	13 (4%)	15 (6%)		2 (6%)	0	0	0	2 (8%)	13 (6%)	10 (7%)	2 (8%)	1 (3%)
Ongoing, unknown	22 (6%)	15 (6%)	··	3 (9%)	2 (25%)	0	0	1 (4%)	12 (6%)	9 (6%)	0	3 (8%)
Sputum	··	··	0·089	··	··	··	··	··	··	··	··	··
Positive	264 (78%)	205 (84%)	··	25 (71%)	7 (88%)	1 (50%)	1 (100%)	16 (67%)	180 (87%)	129 (88%)	17 (71%)	34 (89%)
Negative	68 (20%)	36 (15%)	··	10 (29%)	1 (13%)	1 (50%)	0	8 (33%)	26 (13%)	17 (12%)	6 (25%)	3 (8%)

Data are n (%) or median (IQR). p values show the difference between pan-susceptible and any resistance, obtained with the χ^2^ test (L5 and L6 were excluded and for age the *t* test was used). The category other included the following drug resistances: cycloserine (n=1); ethionamide (n=5); streptomycin (n=9); ethambutol and rifampicin (n=1); ethambutol and streptomycin (n=1); isoniazid and ethionamide (n=14); isoniazid and pyrazinamide (n=1); isoniazid and streptomycin (n=1); ethambutol, isoniazid, and streptomycin (n=1); isoniazid, ethionamide, and streptomycin (n=1); rifampicin, pyrazinamide, streptomycin, and ethionamide (n=1); isoniazid, levofloxacin, moxifloxacin, ofloxacin, para-aminosalicylic acid, and ciprofloxacin (n=1); ethambutol, rifampicin, levofloxacin, moxifloxacin, ofloxacin, ciprofloxacin, and streptomycin (n=1). XDR=extensively drug-resistant. Due to rounding, some group percentage totals are more than 100%.

**Table 2:** Drug resistance results from whole-genome sequencing and local testing by diagnosis concordance and potential consequences for treatment

Drug resistance		n (%)	Local drug susceptibility test diagnosis method			
Based on whole-genome sequencing	Based on local tests		Xpert MTB/RIF*	Culture	Line probe assay	Combination of tests
**Concordance between resistance patterns**						
Total	··	452 (100%)	242/452 (54%)	195/452 (43%)	60/452 (13%)	102/452 (23%)
Pan-susceptible	Pan-susceptible	293 (65%)	196	139	49	53
Monoresistant (3 isoniazid, 2 rifampicin)	3 isoniazid, 2 rifampicin	5 (1%)	0	4	1	0
MDR	MDR	138 (31%)	45	44	8	44
Pre-XDR or XDR	Pre-XDR or XDR	9 (2%)	1	1	2	5
Other (7 streptomycin)	7 streptomycin	7 (2%)	0	7	0	0
**Discordance between resistance patterns**						
Total	··	130 (100%)	35/130 (27%)	55/130 (42%)	9/130 (7%)	46/130 (35%)
Potentially leading to undertreatment	··	34 (26%)	17/130 (13%)	12/130 (9%)	1/130 (1%)	1/130 (1%)
Pan-susceptible	··	0	0	0	0	0
Monoresistant (3 isoniazid)	3 pan-susceptible	3 (2%)	2	1	0	0
MDR	3 pan-susceptible, 1 streptomycin-ethambutol	4 (3%)	2	1	0	1
Pre-XDR or XDR	15 MDR	15 (12%)	5	6	0	4
Other (10 isoniazid-ethionamide, 1 isoniazid-streptomycin, 1 isoniazid-ethionamide-streptomycin)	12 pan-susceptible	12 (9%)	8	4	0	0
Potentially leading to overtreatment	··	31 (24%)	3/130 (2%)	12/130 (9%)	2/130 (2%)	14/130 (11%)
Pan-susceptible	1 isoniazid, 18 MDR, 4 rifampicin	23 (18%)	3	9	2	9
Monoresistant (2 isoniazid)	2 MDR	2 (2%)	0	2	0	0
MDR	3 Pre-XDR or XDR	3 (2%)	0	0	0	3
Pre-XDR or XDR	··	0	0	0	0	0
Other (1 isoniazid-ethionamide, 1 isoniazid-pyrazinamide, 1 isoniazid-ethambutol-streptomycin)	3 MDR	3 (2%)	0	1	0	2
Other discordance	··	65 (50%)	15/130 (12%)	31/130 (9%)	6/130 (1%)	27/130 (4%)
Pan-susceptible	20 ethambutol, monoresistant†, streptomycin	23 (18%)	2	20	0	1
Monoresistant (1 ethambutol, 2 pyrazinamide, 22 rifampicin)	1 pan-susceptible, 2 pan-susceptible, 22 MDR	25 (19%)	6	1	1	20
MDR	1 rifampicin	1 (1%)	0	0	1	0
Pre-XDR or XDR	··	0	0	0	0	0
Other‡		16 (12%)	7	10	5	6
1 cycloserine, 5 ethionamide 2 streptomycin	1 pan-susceptible 5 pan-susceptible 1 pan-susceptible, 1 streptomycin-ethambutol					
3 isoniazid-ethionamide 1 isoniazid-levofloxacin-moxifloxacin-ofloxacin-para-aminosalicylic acid-ciprofloxacin	3 isoniazid 1 isoniazid					
1 ethambutol-rifampicin-levofloxacin-moxifloxacinofloxacin-ciprofloxacin-streptomycin	1 streptomycin					
1 ethambutol-rifampicin 1 ethambutol-streptomycin 1 rifampicin-pyrazinamide-streptomycin-ethionamide	1 MDR 1 MDR 1 MDR					

MDR=multidrug-resistant. XDR=extensively drug-resistant.

* Rifampicin resistance diagnosed with Xpert MTB/RIF was classified as MDR.

† Exact monoresistance is not known.

**Table 3:** Mortality by concordance of local diagnosis and whole-genome sequencing

	Total	Concordant with diagnosis at sites	Discordant with diagnosis at sites			
			Any discordance	Potentially leading to undertreatment	Potentially leading to overtreatment	Other discordance
Resistance based on whole-genome sequencing	63/530 (12%)	44/409 (11%)	19/121 (16%)	8/30 (27%)	1/28 (4%)	10/63 (16%)
Pan-susceptible	18/310 (6%)	16/267 (6%)	2/43 (5%)	0/0	0/20	2/23 (9%)
Any resistance	45/220 (20%)	28/142 (20%)	17/78 (22%)	8/30 (27%)	1/8 (13%)	8/40 (20%)
Monoresistance	6/32 (19%)	2/4 (50%)	4/28 (14%)	0/1	0/2	4/25 (16%)
Isoniazid	1/6 (17%)	1/3 (33%)	0/3	0/1	0/2	0/0
Pyrazinamide	1/2 (50%)	0/0	1/2 (50%)	0/0	0/0	1/2 (50%)
Ethambutol	0/1	0/0	0/1	0/0	0/0	0/1
Rifampicin	4/23 (17%)	1/1 (100%)	3/22 (14%)	0/0	0/0	3/22 (14%)
Polyresistance	39/188 (21%)	26/138 (19%)	13/50 (26%)	8/29 (28%)	1/6 (17%)	4/15 (27%)
Multidrug resistance	24/131 (18%)	23/123 (19%)	1/8 (13%)	1/4 (25%)	0/3	0/1
Pre-XDR or XDR	9/23 (39%)	2/8 (25%)	7/15 (47%)	7/15 (47%)	0/0	0/0
Other	6/34 (18%)	1/7 (14%)	5/27 (19%)	0/9	1/3 (33%)	4/15 (27%)

Analysis based on 530 patients with complete data. The category other discordance includes the following drug resistances: cycloserine (n=1); ethionamide (n=5); streptomycin (n=9); ethambutol and rifampicin (n=1); isoniazid and ethionamide (n=12); isoniazid and pyrazinamide (n=1); ethambutol, isoniazid, and streptomycin (n=1); ethambutol, isoniazid, and streptomycin (n=1); rifampicin, pyrazinamide, streptomycin, and ethionamide (n=1); isoniazid, levofloxacin, moxifloxacin, ofloxacin, para-aminosalicylic acid, and ciprofloxacin (n=1); and ethambutol, rifampicin, levofloxacin, moxifloxacin, ofloxacin, ciprofloxacin, and streptomycin (n=1). XDR=extensively drug-resistant.

## Research in context

### Evidence before this study

Drug-resistant tuberculosis, in particular multidrug-resistant tuberculosis and extensively drug-resistant tuberculosis, is threatening the control of tuberculosis worldwide. WHO has highlighted the need to improve drug susceptibility testing and treatment of drug-resistant tuberculosis, particularly in countries with a high burden of tuberculosis. Whole-genome sequencing (WGS) has the potential to provide resistance profiles for all first-line and second-line anti-tuberculosis drugs and is increasingly replacing other drug resistance testing methods. Yet, the potential of WGS in routine clinical care has not been shown in low-income and middle-income countries, where the burden of drug-resistant tuberculosis is high. We searched PubMed for systematic reviews and original research articles published in any language up to June 29, 2020. We combined terms for “tuberculosis”, “whole-genome sequencing”, and “mortality”. Several validation studies showed that WGS could accurately predict drug resistance; however, we could not find any study showing the potential benefit of WGS-based drug resistance testing on survival.

### Added value of this study

In this study, we compared drug resistance profiles from WGS with routine drug susceptibility test results in seven countries across three continents with a high tuberculosis burden and assessed the effect of undiagnosed drug resistance on mortality. Results from WGS and routine drug susceptibility testing were discordant in 22% of patients. Resistance to isoniazid and rifampicin was accurately identified at local clinics, whereas resistance to ethambutol, pyrazinamide, and second-line drugs was rarely tested locally. Mortality ranged from 6% in patients with pan-susceptible tuberculosis who were appropriately treated to 32% in patients with drug-resistant tuberculosis who were undertreated.

### Implications of all the available evidence

Routine drug susceptibility testing in resource-limited settings with a high tuberculosis burden is often insufficient to inform the prescription of the most effective treatment regimen, which in turn contributes to higher mortality. Our results support the implementation of point-of-care protocols for WGS, ideally directly from sputum to obtain comprehensive drug resistance profiles and facilitate the initiation of personalised and effective treatment regimens.
