# Supplementary material for: Mortality from drug-resistant tuberculosis in high-burden countries comparing routine drug susceptibility testing with whole-genome sequencing: a multicentre cohort study
Source: Lancet Microbe. Author manuscript; Available in PMC 2022 Mar 4. (PMC8896764; doi:10.1016/S2666-5247(21)00044-6)
Supplement: 1 [file NIHMS1720101-supplement-1.pdf]

THE LANCET
Microbe

Supplementary appendix

This appendix formed part of the original submission and has been peer reviewed. We post it as supplied by the authors.

Supplement to: Zürcher K, Reichmuth ML, Ballif M, et al. Mortality from drug-resistant tuberculosis in high-burden countries comparing routine drug susceptibility testing with whole-genome sequencing: a multicentre cohort study. *Lancet Microbe* 2021; published online April 29. https://doi.org/10.1016/S2666-5247(21)00044-6.

Supplementary materials

Zürcher et al. Mortality from drug-resistant tuberculosis in high-burden countries comparing routine drug susceptibility testing with whole-genome sequencing: a multicentre cohort study

Lancet Microbe, 2021

Supplementary Figure 1. Selection of the study population.

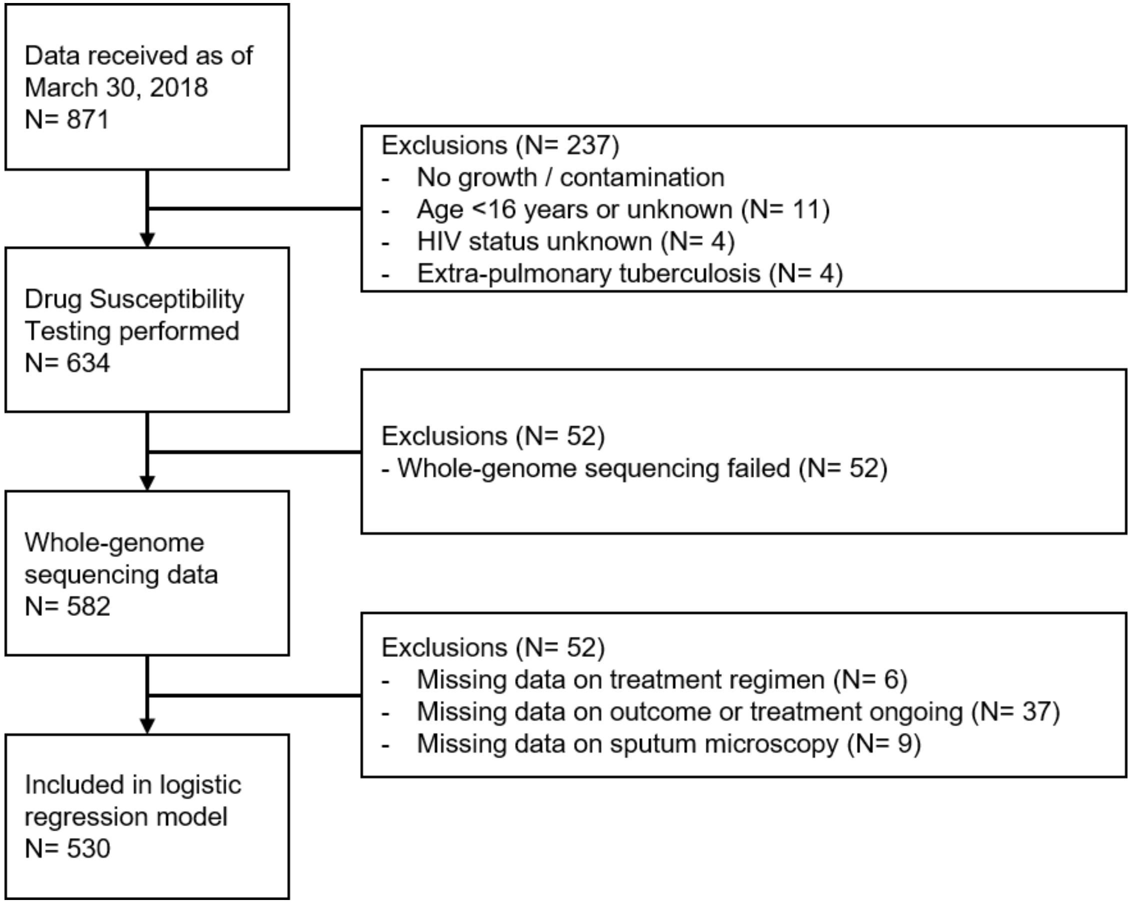

Supplementary Figure 2. Results from sensitivity analysis (using > 0% variant frequency) of four logistic regression models to assess 1) the impact of any drug resistance on mortality, 2) the impact of drug resistance categories on mortality, 3) the impact of diagnosis discordance on mortality, 4) the impact of treatment appropriateness (according to WHO guidelines) on mortality.

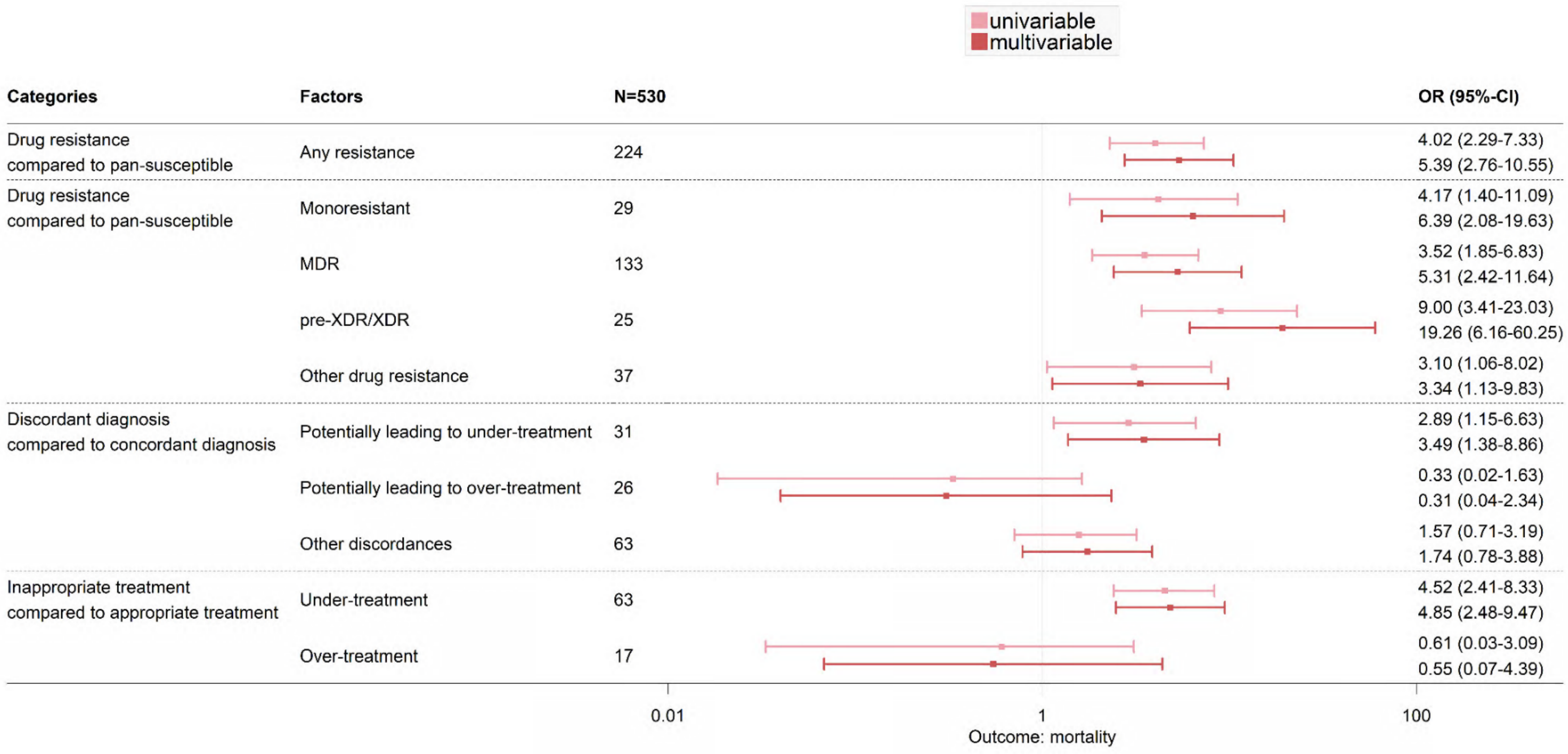

The models were adjusted for sex, age, HIV status, history of tuberculosis, sputum microscopy, and country of participating sites was included as random effect on the intercept.
Abbreviations: OR, odds ratio; CI, confidence interval; N, number; WHO, world health organisation; MDR, multidrug-resistant; Pre-XDR/XDR, pre-/ extensively drug resistant.

Supplementary Figure 3. Results from sensitivity analysis (<u>using 100% variant frequency</u>) of four logistic regression to assess 1) the impact of any drug resistance on mortality, 2) the impact of drug resistance categories on mortality, 3) the impact of diagnosis discordance on mortality, 4) the impact of treatment appropriateness (according to WHO guidelines) on mortality.

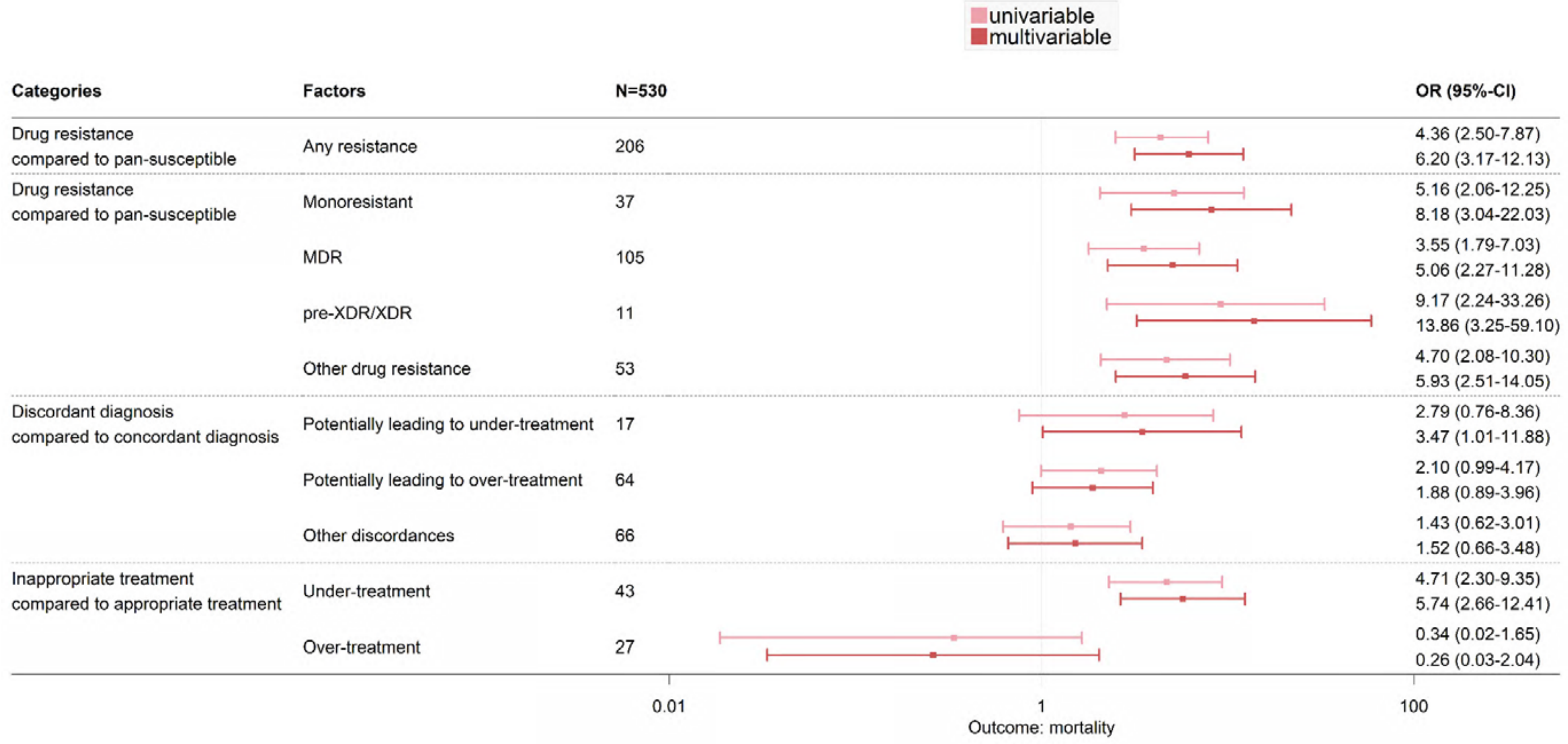

The models were adjusted for sex, age, HIV status, history of tuberculosis, sputum microscopy, and country of participating sites was included as random effect on the intercept.
Abbreviations: OR, odds ratio; CI, confidence interval; N, number; WHO, world health organisation; MDR, multidrug-resistant; Pre-XDR/XDR, pre-/ extensively drug resistant.

Supplementary Figure 4. Results from sensitivity analysis (<u>restricting to drug resistance for which sites could test and ≥ 90% variant frequency</u>) of four logistic regression models to assess 1) the impact of any drug resistance on mortality, 2) the impact of drug resistance categories on mortality, 3) the impact of diagnosis discordance on mortality, 4) the impact of treatment appropriateness (according to WHO guidelines) on mortality.

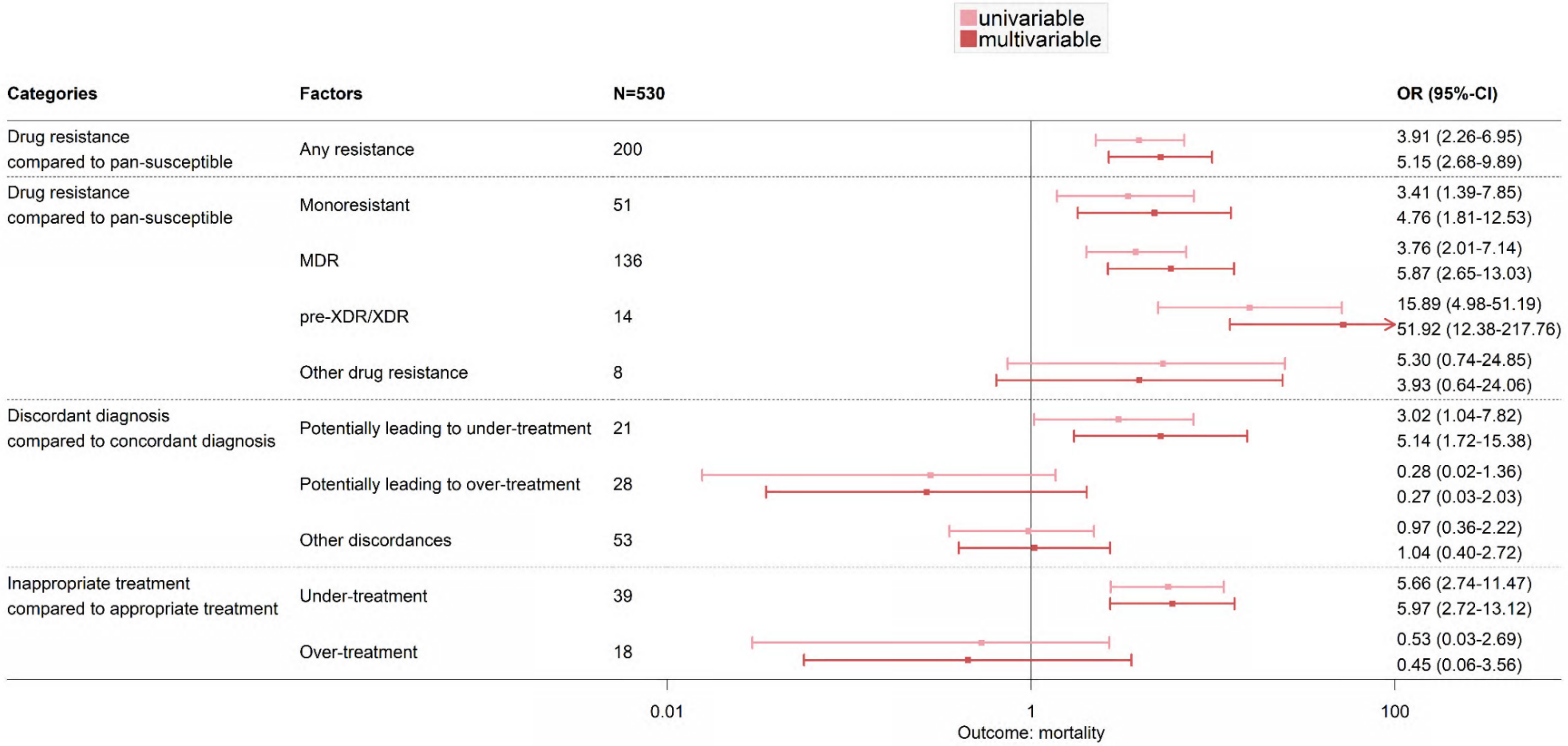

Footnote: The models were adjusted for sex, age, HIV status, history of tuberculosis, sputum microscopy, and country of participating sites was included as random effect on the intercept
Abbreviations: OR, odds ratio; CI, confidence interval; N, number; WHO, world health organisation; MDR, multidrug-resistant; Pre-XDR/XDR, pre-/ extensively drug resistant.

Supplementary Table 1. Drug resistance category potentially leading to under- and over-treatment.

Drug resistance profile obtained with WGS	Drug resistance profile obtained at sites	Drug resistance profile obtained at sites
	**Potentially leading to under-treatment**	**Potentially leading to over-treatment**
Pan-susceptible	-	INH-, RIF-, or PZA- with EMB- resistance, MDR, or pre-XDR/XDR
Resistance to PZA or EMB	-	INH-, RIF-, or PZA- with EMB- resistance, MDR, or pre-XDR/XDR
Resistance to PZA and EMB	Pan-susceptible, PZA-, or EMB- resistance	INH-, RIF- resistance, MDR, or, pre-XDR/XDR
Resistance to INH	Pan-susceptible, PZA-, or EMB- resistance	RIF- resistance, MDR, or pre-XDR/XDR
Resistance to RIF	Pan-susceptible and resistance without RIF	pre-XDR/XDR
Pre-XDR/XDR	All resistance except pre-XDR/XDR	-

Abbreviations: WGS, whole-genome sequencing; INH, isoniazid; RIF, Rifampicin; PZA, pyrazinamide, EMB, ethambutol; MDR, multidrug-resistance; Pre-XDR/XDR, pre-/extensively drug-resistance.

Supplementary Table 2. Treatment appropriateness according to drug resistance profile.

Drug resistance	Number of effective drugs	Appropriate regimen	WHO guidelines
Pan-susceptible	4	First-line	First-line regimen
EMB- without INH- or RIF-resistance	3-4	First-line	First-line regimen
PZA- without INH- or RIF-resistance	3-4	First-line	First-line regimen
INH-resistance	≥ 4	Second-line	(H-INH) + RIF + PZA + EMB + FQ
RIF-resistance	≥ 4	Second-line	MDR regimen
MDR	≥ 4	Second-line	MDR regimen
Pre-XDR/XDR	≥ 4	Second-line	MDR/XDR regimen

Abbreviations: WHO, World Health Organization; INH, isoniazid; H-INH, high-dose isoniazid; PZA, pyrazinamide; EMB, ethambutol; RIF, rifampicin; MDR, multidrug resistance; Pre-XDR/XDR, pre-/ extensively drug resistance.

Supplementary Table 3. Mutant *Mycobacterium tuberculosis* position and number of strains identified locally as sensitive to a given drug, out of number of strains having the corresponding drug-resistance conferring mutation based on WGS.

**Mutations (gene)**	**Drug**	**No. of strains identified locally as sensitive, out of total no. of strains with the drug-resistance conferring mutation based on WGS**
c.-10G>A (*eis*)	KM	1/1 (100%)
c.-11A>C (*pncA*)	PZA	1/1 (100%)
c.-11A>G (*ethA*)	ETH	2/2 (100%)
c.-11C>A (*embA*); p.Met306Ile (*embB*)	EMB	0/1 (0%)
c.-12C>T (*embA*)	EMB	1/1 (100%)
c.-12C>T (*embA*); p.Asp1024Asn (*embB*)	EMB	1/1 (100%)
c.-14C>T (*eis*)	KM	1/1 (100%)
c.-15C>T (*fabG1*)	ETH INH	24/40 (60%)
c.-15C>T (*fabG1*); c.65_65del (*ethA*); p.Ala95Thr (*ethR*)	ETH INH	1/1 (100%)
c.-15C>T (fabG1); c.827_827del (*ethA*)	ETH INH	1/1 (100%)
c.-15C>T (*fabG1*); p.Ala381Pro (*ethA*)	ETH INH	1/1 (100%)
c.-15C>T (*fabG1*); p.Ala95Thr (*ethR*)	ETH INH	7/7 (100%)
c.-15C>T (*fabG1*); p.Ile194Thr (*inhA*)	ETH INH	0/5 (0%)
c.-15C>T (*fabG1*); p.Ile194Thr (*inhA*); p.Ala95Thr (*ethR*)	ETH INH	5/5 (100%)
c.-15C>T (*fabG1*); p.Ile21Thr (*inhA*)	ETH INH	3/6 (50%)
c.-15C>T (*fabG1*); p.Ile21Thr (*inhA*); p.Ala381Pro (*ethA*)	ETH INH	1/1 (100%)
c.-15C>T (*fabG1*); p.Ile21Val (*inhA*)	ETH INH	1/1 (100%)
c.-15C>T (*fabG1*); p.Ser315Gly (*katG*)	ETH INH	1/1 (100%)
c.-15C>T (*fabG1*); p.Ser315Thr (*katG*)	INH ETH	1/7 (14%)
c.-16C>G (*embA*)	EMB	1/1 (100%)
c.-16C>T (*embA*)	EMB	1/1 (100%)
c.-16C>T (*embA*); p.Met306Ile (*embB*)	EMB	1/1 (100%)
c.-16C>T (*embA*); p.Met306Val (*embB*)	EMB	1/1 (100%)
c.-16C>T (*thyX*)	PAS	1/1 (100%)
c.-17G>T (*fabG1*)	ETH INH	2/3 (67%)
c.-17G>T (*fabG1*); p.Ala381Pro (*ethA*)	ETH INH	7/7 (100%)
c.-17G>T (*fabG1*); p.Ser315Thr (*katG*)	INH ETH	1/8 (12%)
c.-48G>A (*ahpC*)	INH	0/1 (0%)
c.-54C>T (*ahpC*)	INH	1/1 (100%)
c.-74G>A (*ahpC*)	INH	1/1 (100%)
c.-8T>A (*fabG1*); p.Ser315Thr (*katG*)	INH	4/7 (57%)
c.-8T>C (*fabG1*)	ETH INH	14/14 (100%)
c.-8T>C (*fabG1*); p.Ser315Thr (*katG*)	INH ETH	8/14 (57%)
c.102_102del (*gid*)	SM	5/7 (71%)
c.1054_1054del (*ethA*)	ETH	1/1 (100%)
c.115_115del (*gid*)	SM	6/7 (86%)
c.1247_1248insT (*katG*); c.-81C>T (*ahpC*)	INH	0/1 (0%)
c.1282_1290del (*rpoB*)	RIF	1/1 (100%)
c.1292_1293del (*ethA*)	ETH	1/1 (100%)
c.139_139del (*pncA*)	PZA	1/1 (100%)
c.140_140del (*ethA*)	ETH	2/2 (100%)
c.171_172insAC (*pncA*)	PZA	1/1 (100%)
c.210_210del (*ethA*)	ETH	1/1 (100%)
c.341_341del (*ethA*)	ETH	2/2 (100%)
c.351_351del (*gid*)	SM	2/2 (100%)
c.389_389del (*pncA*)	PZA	2/2 (100%)
c.391_392insG (*pncA*)	PZA	1/1 (100%)
c.407_408insA (*pncA*)	PZA	1/1 (100%)
c.433_434insGC (*ald*)	CS	1/1 (100%)
c.459_466del (*pncA*)	PZA	1/1 (100%)
c.517_518insG (*pncA*)	PZA	7/7 (100%)
c.597_597del (*ethA*)	ETH	1/1 (100%)
c.626_627del (*ethA*)	ETH	2/2 (100%)
c.639_640del (*ethA*)	ETH	3/3 (100%)
Chromosome:g.2288682_2289080del (*pncA*)	PZA	1/1 (100%)
Chromosome:g.4407912_4408042del (*gid*)	SM	2/2 (100%)
p.Ala146Glu (*pncA*)	PZA	1/1 (100%)
p.Ala146Thr (*pncA*)	PZA	4/4 (100%)
p.Ala28Asp (*pncA*)	PZA	1/1 (100%)
p.Ala381Pro (*ethA*)	ETH	1/1 (100%)
p.Ala90Val (*gyrA*)	CIP LFX MFX OFX	6/6 (100%)
p.Ala95Thr (*ethR*)	ETH	2/2 (100%)
p.Asn138His (*katG*)	INH	0/1 (0%)
p.Asp12Ala (*pncA*)	PZA	3/4 (75%)
p.Asp136Gly (*pncA*)	PZA	1/1 (100%)

p.Asp354Ala (*embB*)	EMB	1/1 (100%)
p.Asp435Phe (*rpoB*)	RIF	0/3 (0%)
p.Asp435Tyr (*rpoB*)	RIF	0/2 (0%)
p.Asp435Tyr (*rpoB*); p.Pro454His (*rpoB*)	RIF	0/1 (0%)
p.Asp435Val (*rpoB*)	RIF	0/42 (0%)
p.Asp494Ala (*gyrB*)	CIP LFX MFX OFX	1/1 (100%)
p.Asp8Ala (*pncA*)	PZA	1/1 (100%)
p.Asp8Asn (*pncA*)	PZA	2/2 (100%)
p.Asp8Gly (pncA)	PZA	1/1 (100%)
p.Asp94Ala (*gyrA*)	CIP LFX MFX OFX	4/5 (80%)
p.Asp94Gly (*gyrA*)	CIP LFX MFX OFX	8/12 (67%)
p.Cys14Arg (*pncA*)	PZA	2/2 (100%)
p.Cys14Gly (*pncA*)	PZA	1/1 (100%)
p.Cys14Trp (*pncA*)	PZA	1/1 (100%)
p.Cys72Arg (*pncA*)	PZA	1/1 (100%)
p.Gln10Arg (*pncA*)	PZA	5/5 (100%)
p.Gln165Pro (*ethA*)	ETH	3/3 (100%)
p.Gln429His (*rpoB*); p.Asp435Phe (*rpoB*)	RIF	0/1 (0%)
p.Gln429His (*rpoB*); p.Asp435Tyr (*rpoB*)	RIF	0/2 (0%)
p.Gln432Leu (*rpoB*)	RIF	0/1 (0%)
p.Gln432Lys (rpoB)	RIF	0/1 (0%)
p.Gln497Arg (*embB*)	EMB	7/8 (88%)
p.Gln497Lys (*embB*)	EMB	2/2 (100%)
p.Gln497Lys (*embB*); p.Asp1024Asn (*embB*)	EMB	1/1 (100%)
p.Glu405Asp (*embB*)	EMB	1/1 (100%)
p.Gly234Glu (*katG*); c.-74G>A (*ahpC*)	INH	0/1 (0%)
p.Gly240Arg (*embR*)	EMB	1/1 (100%)
p.Gly297Val (*katG*)	INH	1/1 (100%)
p.Gly406Ala (*embB*)	EMB	2/2 (100%)
p.Gly406Asp (*embB*)	EMB	3/3 (100%)
p.Gly406Asp (*embB*); p.Asp1024Asn (*embB*)	EMB	1/1 (100%)
p.Gly406Cys (*embB*)	EMB	1/1 (100%)
p.Gly406Ser (*embB*)	EMB	2/2 (100%)
p.Gly406Ser (*embB*); p.His1002Arg (*embB*)	EMB	1/1 (100%)
p.Gly78Asp (*pncA*)	PZA	1/1 (100%)
p.Gly97Cys (*pncA*)	PZA	1/1 (100%)
p.His1002Arg (*embB*)	EMB	1/1 (100%)
p.His445Asn (*rpoB*)	RIF	0/1 (0%)
p.His445Asp (*rpoB*)	RIF	0/16 (0%)
p.His445Gln (*rpoB*)	RIF	0/1 (0%)
p.His445Leu (*rpoB*)	RIF	1/7 (14%)
p.His445Ser (*rpoB*)	RIF	0/2 (0%)
p.His445Ser (*rpoB*); p.Lys446Gln (*rpoB*)	RIF	0/2 (0%)
p.His445Thr (*rpoB*); p.Lys446Gln (*rpoB*)	RIF	0/1 (0%)
p.His445Tyr (*rpoB*)	RIF	0/9 (0%)
p.His51Arg (*pncA*)	PZA	8/8 (100%)
p.His57Tyr (*pncA*)	PZA	2/2 (100%)
p.His71Arg (*pncA*)	PZA	1/1 (100%)
p.His82Arg (*pncA*)	PZA	1/1 (100%)
p.Ile43Thr (*folC*)	PAS	1/1 (100%)
p.Ile491Phe (*rpoB*)	RIF	0/1 (0%)
p.Ile5Ser (*pncA*)	PZA	1/1 (100%)
p.Ile90Ser (*pncA*)	PZA	1/1 (100%)
p.Leu113Arg (*alr*)	CS	3/3 (100%)
p.Leu120Pro (*pncA*)	PZA	2/2 (100%)
p.Leu159Arg (*pncA*)	PZA	1/1 (100%)
p.Leu172Pro (*pncA*)	PZA	1/1 (100%)
p.Leu272Pro (*ethA*)	ETH	1/1 (100%)
p.Leu27Pro (*pncA*)	PZA	1/1 (100%)
p.Leu430Pro (*rpoB*)	RIF	0/7 (0%)
p.Leu430Pro (*rpoB*); p.Asp435Gly (*rpoB*)	RIF	0/1 (0%)
p.Leu430Pro (*rpoB*); p.Ser431Thr (*rpoB*)	RIF	0/1 (0%)
p.Leu452Pro (*rpoB*)	RIF	0/6 (0%)
p.Leu4Ser (*pncA*)	PZA	1/1 (100%)
p.Lys43Arg (*rpsL*)	SM	22/35 (63%)
p.Lys48Thr (*pncA*)	PZA	2/2 (100%)
p.Lys88Arg (*rpsL*)	SM	3/6 (50%)
p.Lys88Gln (*rpsL*)	SM	1/1 (100%)
p.Lys88Thr (*rpsL*)	SM	1/1 (100%)
p.Met1000Arg (*embB*)	EMB	1/1 (100%)
p.Met306Ile (*embB*)	EMB	28/34 (82%)
p.Met306Ile (*embB*); p.Asp328Gly (*embB*)	EMB	1/1 (100%)
p.Met306Ile (*embB*); p.Asp354Ala (*embB*)	EMB	0/1 (0%)
p.Met306Ile (*embB*); p.Gly406Ala (*embB*)	EMB	1/1 (100%)
p.Met306Leu (*embB*)	EMB	2/2 (100%)

p.Met306Val (*embB*)	EMB	27/39 (69%)
p.Phe110Leu (*ethR*)	ETH	2/2 (100%)
p.Phe94Leu (*pncA*)	PZA	1/1 (100%)
p.Pro54Leu (*pncA*)	PZA	1/1 (100%)
p.Ser315Asn (*katG*)	INH	0/2 (0%)
p.Ser315Thr (*katG*)	INH	19/111 (17%)
p.Ser315Thr (*katG*); p.Asp73His (*ahpC*)	INH	0/1 (0%)
p.Ser441Gln (*rpoB*)	RIF	0/1 (0%)
p.Ser447Phe (*gyrB*); p.Asp94Gly (*gyrA*)	CIP LFX MFX OFX	1/1 (100%)
p.Ser450Leu (*rpoB*)	RIF	2/79 (3%)
p.Ser450Leu (*rpoB*); p.Leu527Val (*rpoC*)	RIF	0/2 (0%)
p.Ser450Leu (*rpoB*); p.Phe452Ser (*rpoC*)	RIF	0/1 (0%)
p.Ser450Trp (*rpoB*)	RIF	0/1 (0%)
p.Ser67Pro (*pncA*)	PZA	1/1 (100%)
p.Ser94Ala (*inhA*)	ETH INH	2/2 (100%)
p.Thr168Asn (*pncA*)	PZA	1/1 (100%)
p.Thr400Ala (*rpoB*); p.Ser450Leu (*rpoB*)	RIF	0/1 (0%)
p.Thr76Pro (*pncA*)	PZA	1/1 (100%)
p.Tyr103* (*pncA*)	PZA	1/1 (100%)
p.Tyr319Ser (*embB*)	EMB	5/5 (100%)
p.Val139Ala (*pncA*)	PZA	1/1 (100%)
p.Val170Phe (*rpoB*)	RIF	0/2 (0%)
p.Val170Phe (*rpoB*); p.His445Tyr (*rpoB*)	RIF	0/1 (0%)
p.Val1Ala (*katG*)	INH	1/1 (100%)
r.1401a>g (*rrs*)	AMK CAP KM	9/10 (90%)
r.513c>t (*rrs*)	SM	1/1 (100%)
r.514a>c (*rrs*)	SM	10/11 (91%)
r.517c>t (*rrs*)	SM	2/2 (100%)
r.905c>g (*rrs*)	SM	2/2 (100%)
r.906a>g (*rrs*)	SM	1/3 (33%)
r.907a>t (*rrs*)	SM	1/1 (100%)

Footnote: The mutation with the corresponding gene is according to the Human Genome Variation Society (HGVS) nomenclature. The c. stand for coding DNA reference sequence, p. for protein reference sequence, r. for RNA reference sequence, and the * in the mutation stands for translation termination (stop) codon.

Abbreviations: SM, streptomycin; PAS, para-aminosalicylic-acid; INH, isoniazid; PZA, pyrazinamide; CS, cycloserine; KM, kanamycin; ETH, ethionamide; EMB, ethambutol; RIF, rifampicin; OFX, ofloxacin; CIP, ciprofloxacin; MFX moxifloxacin; LFX, levofloxacin, LZD, linezolid; BDQ, bedaquiline; CFZ, clofazimine; DLM, delamanid; CLR, clarithromycin; AMX, amoxicillin; CM, capreomycin; AM, Amikacin

Supplementary Table 4. Classification of treatment regimens by drug resistance profile.

Drug resistance according to WGS	Total	Treatment compatible with WHO guidelines	Total	Under-treatment	Total	Over-treatment
**Pan-susceptible**	320	320 INH PZA EMB RIF	0		19	1 INH PZA EMB OFX
						2 INH PZA KM EMB MFX CFZ
						1 PZA CS KM EMB LFX
						4 PZA CS KM LFX
						9 PZA KM ETH EMB MFX
						1 SM INH PZA EMB OFX
						1 SM INH PZA EMB RIF
**Monoresistance**	27		8		0	
INH	1	1 PZA CS KM LFX	7	7 INH PZA EMB RIF	0	
PZA	2	2 INH PZA EMB RIF	0		0	
EMB	1	1 INH PZA EMB RIF	0		0	
RIF	23	2 INH PZA KM EMB MFX CFZ	1	1 INH PZA EMB RIF	0	
		2 PZA CS AMK LFX				
		4 PZA CS KM LFX				
		14 PZA KM ETH EMB MFX				
		1 SM INH PZA EMB RIF				
**MDR**	110	51 INH PZA KM EMB MFX CFZ	30	1 CS KM ETH EMB LFX	0	
		2 PAS INH PZA KM ETH EMB MFX LZD BDQ CFZ DLM		15 INH PZA EMB RIF		
		2 PAS PZA CS KM ETH EMB		1 INH PZA KM EMB LFX		
		1 PAS PZA CS KM ETH LFX		1 PZA CS KM ETH EMB LFX		
		1 PAS PZA KM ETH EMB LFX		1 PZA EMB RIF LFX		
		1 PAS PZA KM ETH MFX LZD CFZ		11 PZA KM ETH EMB MFX		
		3 PZA CS AMK LFX				
		1 PZA CS ETH EMB AMK LFX				
		3 PZA CS KM EMB LFX				
		1 PZA CS KM ETH EMB				
		7 PZA CS KM ETH EMB LFX				
		21 PZA CS KM LFX				
		14 PZA KM ETH EMB MFX				
		2 SM INH PZA EMB RIF				
**Pre-XDR/XDR**	12	3 INH PZA KM EMB MFX CFZ	12	1 INH PZA EMB RIF	0	
		2 PAS INH PZA KM ETH EMB MFX LZD BDQ CFZ DLM		7 INH PZA KM EMB MFX CFZ		
		1 PAS PZA CS KM ETH EMB		1 PZA CS KM EMB OFX		
		2 PAS PZA KM ETH MFX LFX LZD BDQ		3 PZA CS KM LFX		
		3 PAS PZA KM ETH MFX LZD CFZ				
		1 PZA CS KM LFX				
**Other:**	21		17		0	
5 ETH		5 INH PZA EMB RIF				
1 CS		1 INH PZA EMB RIF				
9 SM		9 INH PZA EMB RIF				
1 EMB SM		1 INH PZA EMB RIF				

1 EMB RIF LFX MFX OFX CIP SM	1 PZA CS KM LFX	
1 RIF PZA ETH SM	1 PZA KM ETH EMB MFX	
1 INH PZA	1 PZA KM ETH EMB MFX	
1 EMB INH SM	1 PZA KM ETH EMB MFX	
1 EMB RIF	1 PZA CS AMK LFX	
14 INH ETH		14 INH PZA EMB RIF
1 INH LFX MFX OFX PAS CIP		1 INH PZA EMB RIF
1 INH SM		1 INH PZA EMB RIF
1 INH ETH SM		1 INH PZA EMB RIF

Abbreviations: vs, versus; WGS, whole-genome sequencing; WHO, World Health Organization; MDR, multidrug resistance; Pre-XDR/XDR, pre-/ extensively drug resistance; SM, streptomycin; PAS, para-aminosalicylic-acid; INH, isoniazid; PZA, pyrazinamide; CS, cycloserine; KM, kanamycin; ETH, ethionamide; EMB, ethambutol; RIF, rifampicin; OFX, ofloxacin; CIP, ciprofloxacin; MFX moxifloxacin; LFX, levofloxacin, LZD, linezolid; BDQ, bedaquiline; CFZ, clofazimine; DLM, delamanid.

Supplementary Table 5. Results from univariable logistic regression model on the probability of death during tuberculosis treatment.

Category	Number of patients (n=530)	Number of deaths (n=63)	OR (95% CI)
**Gender**			
Men	323	44	1.56 (0.9-2.81)
Women	207	19	1
**Age**			
At diagnosis [IQR]	34 (27-43)	37 (30-47)	1.03 (1-1.05)
**HIV status**			
HIV-positive	218	24	1.15 (0.68-2.01)
HIV-negative	312	39	1
**History of tuberculosis**			
Yes	200	29	1.48 (0.86-2.51)
No	330	34	1
**Sputum**			
Positive	427	53	0.76 (0.35-1.49)
Negative	103	10	1

Abbreviations: IQR, interquartile range; OR, odds ratio; CI: Confidence Interval.

Supplementary Table 6. Available and routinely performed drug resistance testing during the study period and in 2020. Newer drugs are indicated in bold.

**Country**	**Study period**		**2020**	
	**DST tests reported as available**	**Drug resistances that were tested among study participants**	**DST tests reported as available**	**Drug resistances which are routinely tested**
**Ivory Coast**	Culture	INH, RIF	Xpert, LPA (HAIN MTBDRplus), Culture	INH, RIF, EMB, PZA, SM
**DRC**	Xpert, Culture	INH, RIF, PZA, EMB, SM, AMK, MFX	Xpert, Culture	RIF, INH, EMB, PZA, SM, MFX, OFX, LFX
**Kenya**	Xpert	INH, RIF	Xpert, LPA (HAIN MTBDRsl), Culture	RIF
**Nigeria**	Xpert, LPA (HAIN MTBDRplus), Culture	INH, RIF	Xpert, LPA (HAIN MTBDRplus and HAIN MTBDRsl), Culture	INH, RIF, AMK, KM, MFX, OFX, LFX, ETH, PAS, CS, CM, CS, VIOMYCIN
**South Africa**	Xpert, LPA (HAIN MTBDRplus), Culture	INH, RIF, INJ, FQ	Xpert, LPA (HAIN MTBDRplus and HAIN MTBDRsl), Culture	RIF, INH, FQ
**Peru**	Culture	INH, RIF	Xpert, LPA (HAIN MTBDRplus), Culture	RIF, INH, EMB
**Thailand**	Culture	INH, RIF, SM	Xpert, LPA (HAIN MTBDRplus and HAIN MTBDRsl), Culture	INH, RIF, EMB, SM, AMK, MFX, OFX, LFX, ETH, PAS, CS, CFZ, AMX, CIP, CLR, IPM, KAN, **LZD**, MPM, PTO

Abbreviations: SM, streptomycin; PAS, para-aminosalicylic-acid; INH, isoniazid; PZA, pyrazinamide; CS, cycloserine; KM, kanamycin; ETH, ethionamide; EMB, ethambutol; RIF, rifampicin; OFX, ofloxacin; CIP, ciprofloxacin; MFX moxifloxacin; LFX, levofloxacin, LZD, linezolid; BDQ, bedaquiline; CFZ, clofazimine; DLM, delamanid; CLR, clarithromycin; AMX, amoxicillin; CM, capreomycin; IPM, imipenem; AMK, amikacin; MPM, meropenem.

Supplementary Table 7. Sequence reference number and accession number for the 582 *Mycobacterium tuberculosis* strains analysed.

Genome IDF	Accession Nr
G03157	SAMN04276734
G03158	SAMN04276698
G03159	SAMN04276741
G03161	SAMN04276714
G03162	SAMN04276692
G03163	SAMN04276696
G03164	SAMN04276702
G03165	SAMN04276691
G03167	SAMN04276652
G03168	SAMN04276744
G03169	SAMN04276653
G03170	SAMN04276700
G03171	SAMN04276654
G03172	SAMN04276701
G03173	SAMN04276735
G03174	SAMN04276699
G03176	SAMN04276727
G03177	SAMN04276720
G03179	SAMN04276703
G03182	SAMN04276715
G03183	SAMN04276655
G03187	SAMN04276740
G03189	SAMN04276688
G03191	SAMN04276706
G03192	SAMN04276656
G03194	SAMN04276708
G03195	SAMN04276710
G03196	SAMN04276657
G03198	SAMN04276709
G03199	SAMN04276658
G03200	SAMN04276704
G03202	SAMN04276689
G03203	SAMN04276717
G03204	SAMN04276659
G03205	SAMN04276693
G03206	SAMN04276660
G03207	SAMN04276737
G03208	SAMN04276694
G03209	SAMN04276729
G03210	SAMN04276745
G03211	SAMN04276712
G03213	SAMN04276661
G03214	SAMN04276732
G03215	SAMN04276662
G03216	SAMN04276731
G03217	SAMN04276663
G03218	SAMN04276664
G03219	SAMN04276726
G03220	SAMN04276713
G03221	SAMN04276725
G03222	SAMN04276665
G03223	SAMN04276666
G03225	SAMN04276707
G03226	SAMN04276668
G03227	SAMN04276669
G03228	SAMN04276670
G03229	SAMN04276733
G03230	SAMN04276671
G03231	SAMN04276728
G03232	SAMN04276730
G03233	SAMN04276718
G03234	SAMN04276722
G03235	SAMN04276743
G03236	SAMN04276687
G03237	SAMN04276721
G04030	SAMN04276755
G04031	SAMN04276807
G04032	SAMN04276787
G04033	SAMN04276754

G04034	SAMN04276767
G04035	SAMN04276773
G04037	SAMN04276818
G04038	SAMN04276792
G04039	SAMN04276800
G04040	SAMN04276779
G04041	SAMN04276748
G04042	SAMN04276830
G04043	SAMN04276822
G04046	SAMN04276825
G04047	SAMN04276823
G04048	SAMN04276836
G04049	SAMN04276797
G04050	SAMN04276798
G04051	SAMN04276806
G04052	SAMN04276811
G04053	SAMN04276815
G04054	SAMN04276771
G04055	SAMN04276824
G04056	SAMN04276808
G04057	SAMN04276757
G04059	SAMN04276821
G04060	SAMN04276834
G04061	SAMN04276799
G04062	SAMN04276776
G04063	SAMN04276831
G04064	SAMN04276796
G04065	SAMN04276829
G04066	SAMN04276753
G04067	SAMN04276750
G04068	SAMN04276751
G04069	SAMN04276802
G04070	SAMN04276820
G04071	SAMN04276804
G04072	SAMN04276810
G04073	SAMN06251200
G04074	SAMN04276749
G04075	SAMN04276752
G04076	SAMN04276782
G04077	SAMN04276778
G04078	SAMN04276770
G04079	SAMN04276780
G04081	SAMN04276769
G04082	SAMN04276763
G04084	SAMN04276794
G04086	SAMN04276765
G04087	SAMN04276777
G04088	SAMN04276764
G04089	SAMN04276774
G04090	SAMN04276772
G04092	SAMN04276775
G04093	SAMN04276801
G04094	SAMN04276813
G04095	SAMN04276762
G04096	SAMN04276786
G04097	SAMN04276809
G04098	SAMN04276758
G04100	SAMN04276814
G04102	SAMN04276766
G04104	SAMN04276841
G04105	SAMN04276833
G04106	SAMN04276835
G04107	SAMN04276840
G04108	SAMN04276812
G04109	SAMN04276826
G04110	SAMN04276837
G04111	SAMN04276790
G04113	SAMN04276832
G04114	SAMN04276756
G04115	SAMN04276817
G04116	SAMN04276768
G04117	SAMN04276784
G04120	SAMN04276793
G04122	SAMN04276839
G04123	SAMN04276828

G04124	SAMN04276819
G04169	SAMN04276805
G04316	SAMN04276679
G04317	SAMN04276680
G04318	SAMN04276681
G04319	SAMN04276682
G04320	SAMN06251197
G04322	SAMN04276683
G05010	SAMN04521938
G05011	SAMN04522015
G05012	SAMN04522025
G05013	SAMN04522026
G05014	SAMN04522017
G05015	SAMN04522021
G05017	SAMN04522023
G05018	SAMN04522031
G05020	SAMN04522029
G05021	SAMN04522028
G05022	SAMN04522033
G05024	SAMN04522013
G05025	SAMN04521995
G05026	SAMN04521996
G05028	SAMN04521998
G05032	SAMN04522002
G05034	SAMN04522004
G05035	SAMN04522005
G05036	SAMN04522006
G05037	SAMN04522007
G05038	SAMN04522008
G05039	SAMN04522024
G05041	SAMN04522022
G05043	SAMN04522011
G05044	SAMN04522016
G05045	SAMN04522027
G05046	SAMN04521939
G05047	SAMN04521940
G05048	SAMN04521941
G05051	SAMN04521944
G05052	SAMN04521945
G05053	SAMN04521946
G05054	SAMN04521947
G05056	SAMN04521949
G05058	SAMN04521951
G05059	SAMN04521952
G05060	SAMN04521953
G05061	SAMN04521954
G05063	SAMN04521956
G05064	SAMN04521957
G05065	SAMN04521958
G05066	SAMN04521959
G05067	SAMN04521960
G05068	SAMN04521961
G05069	SAMN04521962
G05070	SAMN04521963
G05071	SAMN04521964
G05072	SAMN04521965
G05074	SAMN04521967
G05075	SAMN04521968
G05076	SAMN04521969
G05077	SAMN04521970
G05078	SAMN04521971
G05079	SAMN04521972
G05080	SAMN04521973
G05081	SAMN04521974
G05082	SAMN04521975
G05083	SAMN04521976
G05084	SAMN04521977
G05085	SAMN04521978
G05086	SAMN04521979
G05087	SAMN04521980
G05088	SAMN04521981
G05089	SAMN04521982
G05090	SAMN04521983
G05091	SAMN04521984
G05092	SAMN04521985

G05093	SAMN04521986
G05094	SAMN04521987
G05095	SAMN04522009
G05096	SAMN04521988
G05097	SAMN04521989
G05098	SAMN04521990
G05099	SAMN04521991
G05100	SAMN04521992
G05101	SAMN04521993
G05102	SAMN04521994
G05103	SAMN04522010
G05106	SAMN04522071
G05109	SAMN04522036
G05110	SAMN04522038
G05112	SAMN04522041
G05114	SAMN04522048
G05122	SAMN04522052
G05123	SAMN04522053
G05125	SAMN06251204
G05127	SAMN04522057
G05132	SAMN04522061
G05137	SAMN04522069
G05138	SAMN04522068
G05140	SAMN04522075
G05144	SAMN04522078
G05146	SAMN06251205
G05149	SAMN06251206
G05150	SAMN04522098
G05152	SAMN04522081
G05156	SAMN04522085
G05158	SAMN04522035
G05160	SAMN04522093
G05162	SAMN04522095
G05163	SAMN04522125
G05164	SAMN04522126
G05165	SAMN04522097
G05168	SAMN04522100
G05169	SAMN04522114
G05171	SAMN04522101
G05172	SAMN04522102
G05173	SAMN04522103
G05174	SAMN04522104
G05175	SAMN04522107
G05177	SAMN04522108
G05178	SAMN04522106
G05179	SAMN04522111
G05180	SAMN04522109
G05181	SAMN04522112
G05182	SAMN04522110
G05184	SRR7517725
G05187	SAMN04522118
G05189	SAMN04522120
G05190	SAMN04522121
G05194	SAMN04522051
G05196	SAMN04522087
G05198	SAMN04522089
G05199	SAMN04522090
G05203	SAMN04522128
G05204	SAMN04522129
G05207	SAMN04522127
G05209	SAMN04522049
G07408	SRR7517680
G07409	SAMN06251226
G07410	SAMN06251221
G07411	SAMN06251255
G07412	SAMN06251250
G07413	SAMN06251253
G07414	SAMN06251256
G07415	SAMN06251203
G07416	SAMN06251243
G07417	SAMN06251225
G07418	SRR7517681
G07422	SAMN06251222
G07424	SAMN06251228
G07425	SAMN06251223

G07426	SAMN06251224
G07427	SAMN06251207
G07428	SAMN06251246
G07430	SAMN06251247
G07432	SAMN06251230
G07435	SAMN06251234
G07438	SAMN06251242
G07440	SAMN06251244
G07442	SAMN06251227
G07443	SAMN06251252
G07444	SRR7517678
G07446	SRR7517679
G07447	SRR7517684
G07448	SRR7517685
G07450	SRR7517683
G07452	SRR7517687
G07453	SRR7517780
G07454	SRR7517779
G07455	SAMN06251251
G07456	SAMN06251254
G07457	SAMN06251235
G07459	SAMN06251245
G07460	SAMN06251249
G07461	SAMN06251202
G07462	SAMN06251208
G07463	SAMN06251209
G07464	SAMN06251210
G07465	SAMN06251211
G07466	SAMN06251212
G07467	SAMN06251213
G07468	SAMN06251214
G07469	SAMN06251215
G07470	SAMN06251219
G07471	SAMN06251220
G07472	SAMN06251216
G07473	SAMN06251217
G07474	SAMN06251218
G07479	SAMN06251201
G07480	SRR7517778
G07482	SRR7517777
G07486	SRR7517784
G07487	SAMN06251198
G07488	SRR7517783
G07490	SAMN06251199
G07491	SAMN06251240
G07492	SAMN06251248
G07493	SAMN06251237
G08154	SRR7517781
G08155	SRR7517776
G08156	SRR7517775
G08370	SAMN06251193
G08371	SAMN06251194
G08372	SAMN06251195
G08373	SAMN06251196
G08747	SRR7517801
G08799	SRR7517834
G08800	SRR7517831
G08801	SRR7517832
G08802	SRR7517829
G08804	SRR7517827
G08805	SRR7517828
G08806	SRR7517835
G08807	SRR7517836
G08808	SRR7517812
G08809	SRR7517811
G08810	SRR7517810
G08811	SRR7517809
G08812	SRR7517816
G08813	SRR7517815
G08814	SRR7517814
G08815	SRR7517813
G08818	SRR7517792
G08819	SRR7517793
G08820	SRR7517794
G08821	SRR7517795

G08822	SRR7517788
G08823	SRR7517789
G08824	SRR7517790
G08825	SRR7517791
G08827	SRR7517787
G08828	SRR7517768
G08829	SRR7517767
G08830	SRR7517770
G08831	SRR7517769
G08832	SRR7517772
G08834	SRR7517774
G08835	SRR7517773
G08836	SRR7517760
G19889	SAMEA6797830
G19890	SAMEA6797831
G19891	SAMEA6797832
G19892	SAMEA6797833
G19894	SAMEA6797806
G19895	SAMEA6797834
G19896	SAMEA6797835
G19897	SAMEA6797836
G19900	SAMEA6797823
G19904	SAMEA6797816
G19905	SAMEA6797837
G19906	SAMEA6797838
G19912	SAMEA6797840
G19913	SAMEA6797811
G19914	SAMEA6797798
G19915	SAMEA6797841
G19916	SAMEA6797819
G19917	SAMEA6797842
G19918	SAMEA6797843
G19920	SAMEA6797820
G19922	SAMEA6797844
G19923	SAMEA6797804
G19924	SAMEA6797817
G19925	SAMEA6797845
G19926	SAMEA6797801
G19927	SAMEA6797846
G19928	SAMEA6797807
G19929	SAMEA6797825
G19930	SAMEA6797815
G19931	SAMEA6797847
G19932	SAMEA6797848
G19933	SAMEA6797849
G19934	SAMEA6797850
G19936	SAMEA6797851
G19938	SAMEA6797824
G19939	SAMEA6797852
G19940	SAMEA6797853
G19943	SAMEA6797821
G19952	SAMEA6797813
G19954	SAMEA6797814
G19955	SAMEA6797854
G19956	SAMEA6797855
G19957	SAMEA6797856
G19958	SAMEA6797857
G19959	SAMEA6797858
G19961	SAMEA6797859
G19962	SAMEA6797860
G19963	SAMEA6797861
G19964	SAMEA6797862
G19968	SAMEA6797863
G19973	SAMEA6797864
G19974	SAMEA6797865
G19975	SAMEA6797826
G19980	SAMEA6797827
G19981	SAMEA6797866
G19982	SAMEA6797867
G19983	SAMEA6797868
G19984	SAMEA6797805
G19985	SAMEA6797869
G19986	SAMEA6797870
G19987	SAMEA6797871
G19988	SAMEA6797872

G19989	SAMEA6797873
G19990	SAMEA6797874
G19991	SAMEA6797794
G19992	SAMEA6797875
G19993	SAMEA6797876
G19994	SAMEA6797877
G19995	SAMEA6797796
G19996	SAMEA6797878
G19997	SAMEA6797879
G19998	SAMEA6797799
G19999	SAMEA6797795
G20001	SAMEA6797880
G20005	SAMEA6797881
G20007	SAMEA6797882
G20009	SAMEA6797884
G20012	SAMEA6797885
G20014	SAMEA6797886
G20017	SAMEA6797887
G20019	SAMEA6797888
G20021	SAMEA6797889
G20022	SAMEA6797890
G20023	SAMEA6797891
G20034	SAMEA6797892
G20048	SAMEA6797893
G20050	SAMEA6797894
G20082	SAMEA6797904
G20084	SAMEA6797906
G20085	SAMEA6797907
G20086	SAMEA6797810
G20087	SAMEA6797908
G20089	SAMEA6797909
G20091	SAMEA6797808
G20099	SAMEA6797913
G20100	SAMEA6797914
G21714	SAMN14123454
G21720	SAMN14123457
G21726	SAMEA6797921
G21727	SRR11098560
G21733	SAMEA6797922
G21738	SAMN1412345
G21741	SAMN14123455
G21876	SAMEA6797928
G21877	SAMEA6797929
G21881	SAMEA6797931
G21887	SAMEA6797802
G21889	SAMEA6797933
G21891	SAMEA6797935
G21895	SAMEA6797936
G21914	SAMEA6797800
G21922	SAMEA6797822
G21932	SAMEA6797948
G21939	SAMEA6797952
G21940	SAMEA6797953
G21941	SAMEA6797954
G21950	SAMEA6797812
G21968	SAMEA6797803
G21969	SAMEA6797964
G21975	SAMEA6797809
G22164	SAMEA6797967
G22167	SAMEA6797968
G22172	SAMEA6797970
G22174	SAMEA6797971
G22177	SAMEA6797973
G22178	SAMEA6797974
G22181	SAMEA6797976
G22188	SAMEA6797978
G22192	SAMEA6797797
G22193	SAMEA6797980
G22196	SAMEA6797981
G22203	SAMEA6797982
G08795	SAMN15098172
G08796	SAMN15098173
G08798	SAMN15098174
G19893	SAMN15098175
G19899	SAMN15098176

G19901	SAMN15098177
G19902	SAMN15098178
G19903	SAMN15098179
G19908	SAMN15098180
G19909	SAMN15098181
G19911	SAMN15098182
G19919	SAMN15098183
G19935	SAMN15098184
G19937	SAMN15098185
G19941	SAMN15098186
G19945	SAMN15098187
G19946	SAMN15098188
G19950	SAMN15098189
G19953	SAMN15098190
G19960	SAMN15098191
G19965	SAMN15098192
G19967	SAMN15098193
G19970	SAMN15098194
G19971	SAMN15098195
G19972	SAMN15098196
G20029	SAMN15098197
G20035	SAMN15098198
G20036	SAMN15098199
G20037	SAMN15098200
G20038	SAMN15098201
G20054	SAMN15098202
G20056	SAMN15098203
G20058	SAMN15098204
G20062	SAMN15098205
G20063	SAMN15098206
G20064	SAMN15098207
G20065	SAMN15098208
G20066	SAMN15098209
G20067	SAMN15098210
G20068	SAMN15098211
G20069	SAMN15098212
G20088	SAMN15098213
G20090	SAMN15098214
G20092	SAMN15098215
G20093	SAMN15098216
G21716	SAMN15098217
G21717	SAMN15098218
G21719	SAMN15098219
G21721	SAMN15098220
G21723	SAMN15098221
G21724	SAMN15098222
G21729	SAMN15098223
G21731	SAMN15098224
G21732	SAMN15098225
G21743	SAMN15098226
G21744	SAMN15098227
G21905	SAMN15098228
G21919	SAMN15098229
G22156	SAMN15098230
G22157	SAMN15098231
G22159	SAMN15098232
G22189	SAMN15098233
G22190	SAMN15098234
G22194	SAMN15098235
G22197	SAMN15098236
G22198	SAMN15098237
G22200	SAMN15098238
G22201	SAMN15098239

Supplementary Table 8. List of collaborators

First Name	Surname
Alash’le	Abimiku
Anchalee	Avihingsanon
Marie	Ballif
Guido	Bloemberg
Erik C.	Boettger
Sonia	Borrell
E. Jane	Carter
Jimena	Collantes
Helen	Cox
Lameck	Diero
Matthias	Egger
Nicholas	Ezati
Lukas	Fenner
Sebastien	Gagneux
Joachim	Gnokoro
Eduardo	Gotuzzo
Rico	Hömke
Robin	Huebner
Feldmann	Julia
Naruporn	Kasipong
Kamon	Kawkitinarong
Peter	Keller
Olivier	Marcy
Eugène	Messou
Kouassi	N'Guessan
Tor	Petersen
Martina	Reichmuth
Miriam	Reinhard
Neesha	Rockwood
Annette	Sohn
Elizabeth	Streicher
Martine	Tabala
Robin	Warren
Landry	Wenzi
Robert J.	Wilkinson
Marcel	Yotebieng
Carlos	Zamudio
Kathrin	Zürcher
